# The genetic basis of adaptation in phenology in an introduced population of Black Cottonwood (*Populus trichocarpa*, Torr. & Gray)

**DOI:** 10.1186/s12870-021-03103-5

**Published:** 2021-07-02

**Authors:** Rami-Petteri Apuli, Thomas Richards, Martha Rendón-Anaya, Almir Karacic, Ann-Christin Rönnberg-Wästljung, Pär K. Ingvarsson

**Affiliations:** 1 Linnean Centre for Plant Biology, Department of Plant Biology, Uppsala BioCenter, Swedish University of Agricultural Science, Uppsala, Sweden; 2 Plant Ecology and Evolution, Department of Ecology and Genetics, Evolutionary Biology Centre, Uppsala University, Uppsala, Sweden; 3 Institute for Crop Production Ecology, Swedish University of Agricultural Science, Uppsala, Sweden

**Keywords:** *Populus trichocarpa*, Phenology, Local adaptation, Bud burst, Leaf senescence, Introduced population

## Abstract

**Background:**

Entering and exiting winter dormancy present important trade-offs between growth and survival at northern latitudes. Many forest trees display local adaptation across latitude in traits associated with these phenology transitions. Transfers of a species outside its native range introduce the species to novel combinations of environmental conditions potentially requiring different combinations of alleles to optimize growth and survival. In this study, we performed genome wide association analyses and a selection scan in a *P. trichocarpa* mapping population derived from crossings between clones collected across the native range and introduced into Sweden. GWAS analyses were performed using phenotypic data collected across two field seasons and in a controlled phytotron experiment.

**Results:**

We uncovered 584 putative candidate genes associated with spring and autumn phenology traits as well as with growth. Many regions harboring variation significantly associated with the initiation of leaf shed and leaf autumn coloring appeared to have been evolving under positive selection in the native environments of *P. trichocarpa*. A comparison between the candidate genes identified with results from earlier GWAS analyses performed in the native environment found a smaller overlap for spring phenology traits than for autumn phenology traits, aligning well with earlier observations that spring phenology transitions have a more complex genetic basis than autumn phenology transitions.

**Conclusions:**

In a small and structured introduced population of *P. trichocarpa*, we find complex genetic architectures underlying all phenology and growth traits, and identify multiple putative candidate genes despite the limitations of the study population.

**Supplementary Information:**

The online version contains supplementary material available at 10.1186/s12870-021-03103-5.

## Background

At northern latitudes winter conditions are unfavorable to active plant growth through a combination of low temperatures, frosts, and light conditions leading perennial plants to avoid these conditions by entering winter dormancy (hereafter dormancy) [1]. Transitions from active growth to dormancy and from dormancy to active growth are controlled by different environmental cues where the transition to dormancy is primarily induced by changes in photoperiod [2] or light quality [3], while the release of dormancy is induced by prolonged exposure to low temperatures followed by increasing temperatures reactivating growth [4]. Incorrect timing of phenology transitions is known to result in loss of potential growth through extended dormancy or loss of realized growth in the form of damage to important tissues such as meristems and leaves from exposure to unfavorable conditions or even death. Dormancy hence represents an important life history trade-off between growth and survival. Maladapted individuals are likely to suffer lowered reproductive success and/or biomass production, both of which may have large ecological and economic repercussions [5].

Populations of widespread species often display signatures of phenotypic and genetic adaptation to their native environments, even in species with considerable gene flow between the populations [6]. This phenomenon, known as local adaptation, often arises from positive selection [7], which leaves distinct and detectable signatures across the genome [8, 9]. The strength of selection along with local rates of recombination and gene flow are the major determining factors of the extent and magnitude of signatures of selection [9, 10]. Furthermore, associations between segregating polymorphisms in the genomes of individuals and their phenotypes or measures of their natal environment can be explored by performing genome wide association studies (GWAS) [11]. Local adaptation to climate and photoperiod has been observed in a large number of species with wide South-North distribution ranges including *Arabidopsis thaliana* [7], *Picea abies* [12] and *Populus tremula* [13]. Empirical studies suggest that the genetic basis of local adaptation can be highly polygenic, where a majority of the loci and alleles conferring local adaptation have small effects [14] although large effect loci have been observed in some systems (e.g. [15]). Due to the polygenic nature of these traits, the genetic architecture of local adaptation to climate can be very diverse among even closely related species, despite the adaptation being driven by very similar environmental conditions (e.g. [15, 16]).

Species within the genus *Populus* are deciduous, early succession trees with wide distributions across the northern hemisphere, spanning from the equator to the northern limits of tree growth. The rapid growth rate and ability to generate natural clones [17, 18] has spurred economic interest in the genus [17], while many of the species in the genus are also considered keystone species in their natural habitats [19]. *Populus* species are frequently utilized in biomass production for forest industry, even outside of their natural distribution ranges [20]. In northern Europe, biomass production with *Populus* species is an underutilized option due to the phenological maladaptation of commercially bred varieties [21]. Commercial interest thus exists for adapting non-native *Populus* species to growth under northern European conditions, but the required genetic resources and understanding of relevant traits for such an undertaking are lacking [22]. Black cottonwood (*Populus trichocarpa*) is a deciduous tree native to North America with continuous distribution in western and northwest North America from California to Alaska. The species has been thoroughly studied in its natural range and has been found to display signatures of local adaptation to climate and photoperiod across its natural range [16, 23]. Introduction of the species to novel conditions such as those characterizing northern Sweden allow further exploration of the genetic architecture of these traits, and have the potential to reveal novel genes associated with them and comparisons with the results and signatures of selection from the natural range present an opportunity to uncover further details of adaptation, and potential constraints of it in the novel conditions, in these traits. Finally, exploration of the adaptive potential [22] and its genetic basis in small populations resulting from introductions will also offer insight into how to perform cost-effective breeding in forest trees in terms of breeding population size.

In this study, we perform a field trial and a controlled environment phytotron trial to collect data on spring and autumn phenology and growth traits in *P. trichocarpa*. We use the data to dissect the genetic basis of these traits using both population genomic approaches and genome-wide association studies. We compare candidate genes identified across two successive years and between field and phytotron grown plants, in order to identify genes that appear to have reliable effects on different phenology traits. Finally, we compare candidate genes we identified with results from earlier association studies performed in the natural range of *P. trichocarpa* to contrast the genetic control of phenology traits under native and novel environments.

## Results

### Phenotypic variation and heritability

**Fig. 1:**
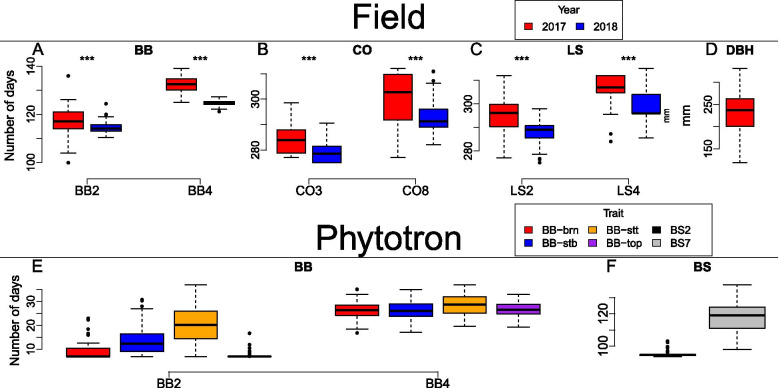
Phenotypes of all study traits. **a** Bud burst (BB), **b** Autumn coloring (CO), **c** Leaf shed (LS), **d** Diameter at breast height (DBH) in the field and (**e**) bud burst and (**f**) bud set (BS) in phytotron. In the box plots, the box spans the first to the third quartile and the median is marked as the thick black line. The whiskers span at most 1.5 times the length of the box with any values further away than that being marked as outliers

All traits (see List of Abbreviations) display variation both within and between years, though BB2-top (Fig. 1E) and BS2 (Fig. 1F) are noticeably less variable than the other traits (Fig. 1). In the field traits there are highly significant (*p *< 0.001) differences between years 2017 and 2018. All chosen traits with the exception of BB2-top and BS2 had an appreciable level of both broad and narrow sense heritability (H^2^ > 0.4, h^2^ > 0.15) with only BB2-top having narrow sense heritability less than 0.05 and BS2 having broad sense heritability below 0.25. There were signs of overfitting for CO8-18 and LS5-18 of the field traits and BS2, BS7, BB2-top, BB4-top, BB2-brn, BB4-brn and BB2-stt, which all were potentially overfitted in the model for BLUP -estimation for calculating the broad sense heritability estimations, as the values approached a singularity fit (Table S7). None of the traits could be considered to be identical, though BB2-stt and BB4-stt, BB4-stt and BB4-stb, and CO3-17 and CO8-17 all had high correlations (r^2^ > 0.8) (Fig. S2).

**Fig. 2:**
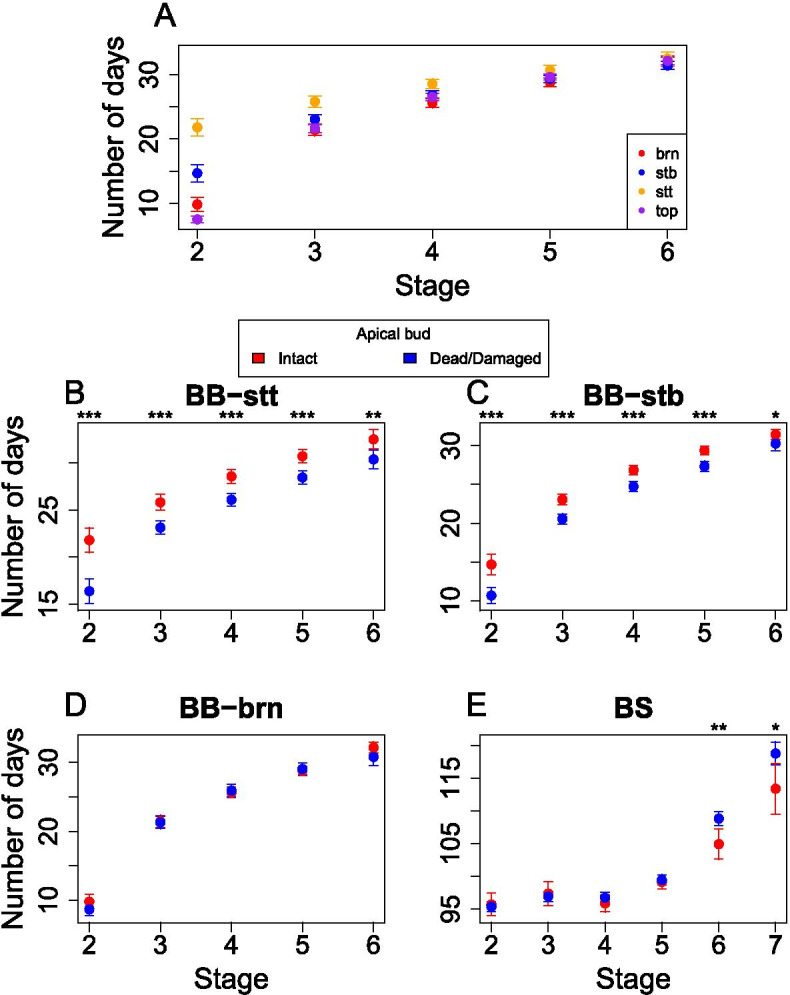
The mean of number days and 95% confidence interval to reach a stage (x-axis) for the phytotron traits. **a** The four different bud types under intact apical bud, **b** the stem top buds (BB-stt) under intact (red) and damaged/dead (blue) apical bud, **c** the stem bottom buds (BB-stb) under intact and damaged/dead apical bud, **d** the branch buds (BB-brn) under intact and damaged/dead apical bud and **e** bud set (BS) under intact and damaged/dead apical bud

The initiation of bud burst was visibly different between the different parts of the plant in the phytotron experiment (brn, stb, stt and top). However, these differences largely disappeared by the 3^rd^ stage for brn, stb and top, while stt remained different until the 4^th^ stage. At stage 5, the different parts of the plants were all behaving in a similar fashion (Fig. 2A). On 1^st^ of August 2018, the phytotron equipment malfunctioned during the first simulated winter, before the spring of the second season, causing the simulated winter temperatures of 4 °C (Table S5) to shift to as high as 21 °C for a few hours, causing early bud flush and subsequent bud damage in some of the cuttings once the winter conditions were restored. When individuals with a damaged or absent apical bud were compared, bud burst timing of both stt (Fig. 2B) and stb (Fig. 2C) were significantly different whereas branches showed no difference (Fig. 2D). A significant difference was also seen in the latter stages of bud set (Fig. 2E).

### Summary of Lindley scores and candidate genes

**Fig. 3:**
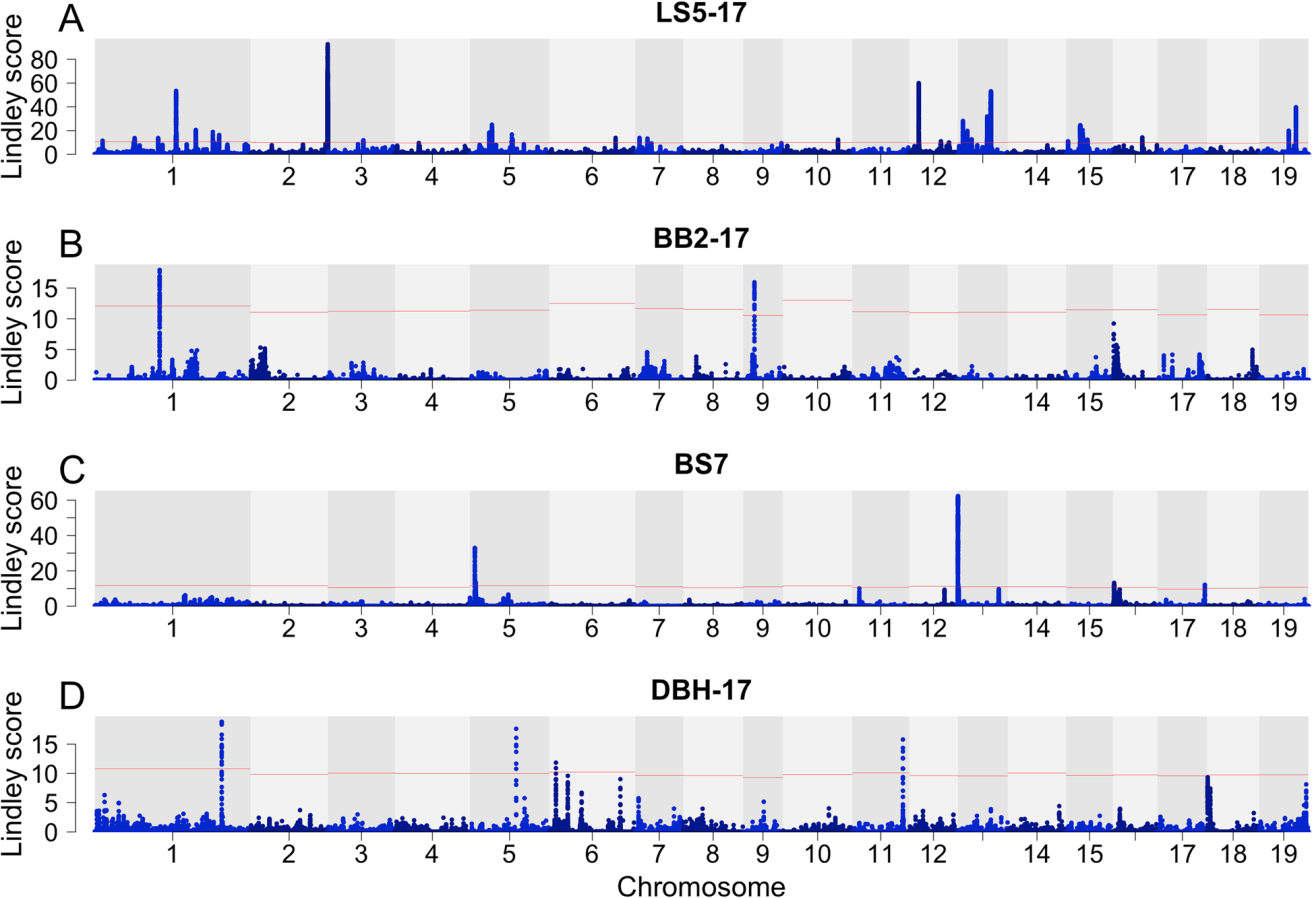
Manhattan plot of the Lindley score for four example traits. **a** Leaf shed completion in 2017 (LS5-17), **b** bud set initiation (BS2), **c** bud burst initiation in the apical bud (BB2-top) and **d** diameter at breast height (DBH-17). The red lines denote the chromosome-specific threshold of significance

The Lindley score method located between 0 and 80 significant slopes for the different traits, yielding overall 250 significant slopes (Table 1, see also Figs. S3 and S4 for traditional Manhattan and quantile–quantile plots). Autumn phenology traits LS5-17 (Fig. 3A), LS2-17 (Fig. 3B) and CO3-17 had the highest numbers of significant slopes with 80, 25 and 21, respectively, followed by BB4-top with 20 significant slopes. Finally, LS5-18, BB4-stb and BB4-brn had 14, 13 and 10 significant slopes respectively. The remainder of the traits, including BS7 (Fig. 3C), had nine or less significant slopes, with no significant slopes identified for BB2-brn (Table 1). The growth trait (DBH-17) had 4 significant slopes (Fig. 3D).

**Table 1:** Numbers of significant slopes and candidate genes (within 10 kbp of significant slope) for each of our 23 chosen autumn phenology, spring phenology and lifetime growth field and phytotron traits

Site	Trait	Significant slopes	Genes
Field	BB2-17	1	2
Field	BB2-18	5	10
Phytotron	BB2-brn	0	0
Phytotron	BB2-stb	5	9
Phytotron	BB2-stt	5	22
Field	BB4-17	9	28
Field	BB4-18	6	21
Phytotron	BB4-brn	10	28
Phytotron	BB4-stb	13	30
Phytotron	BB4-stt	2	5
Phytotron	BB4-top	20	54
Phytotron	BS7	6	30
Field	CO3-17	21	57
Field	CO3-18	7	27
Field	CO8-17	6	23
Field	CO8-18	7	14
Field	DBH-17	4	9
Field	LS2-17	25	53
Field	LS2-18	4	16
Field	LS5-17	80	101
Field	LS5-18	14	45
	Total	250	584

Between 0 and 101 genes were located within 10 kbp of the significant slopes across all traits with 584 unique genes included overall (Table 1). GO-term enrichment analysis for these genes yielded 41 enriched terms across 8 of our traits after multiple test correction. Of these enrichments, 20 were in the category biological process, 13 in cellular component and 8 in molecular function. Overall, 5 out of the 13 significantly enriched cellular components were membranes (Table S8).

### Signatures of positive selection and GO-term enrichment

**Fig. 4:**
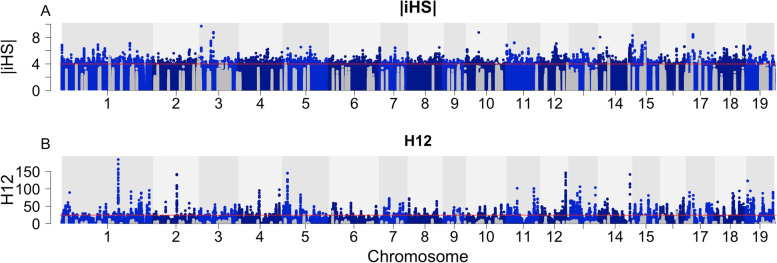
Manhattan plot of the two selection scans. **a** Absolute iHS and **b** H12 selection estimates. The gray dots are markers within 10 kbp of significant slopes

**Fig. 5:**
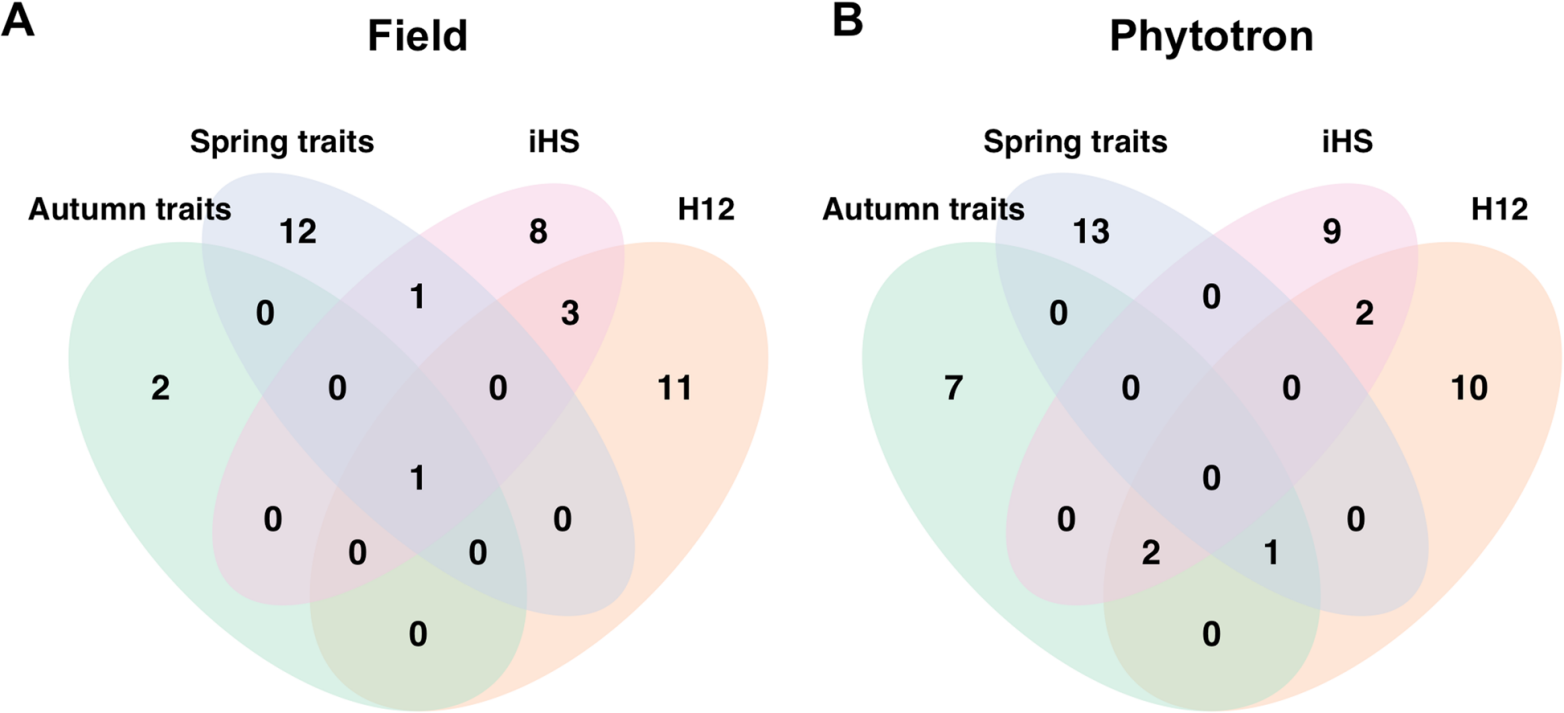
Enriched GO -terms for autumn and spring phenology and the selection estimates iHS and H12. **a** Field and **b** Phytotron

One hundred eight,106 SNPs were located within 10 kbp of the significant slopes and 189 were located in the top 0.1 percentile in the iHS selection scan (Fig. 4A). Similarly, 120,914 SNPs (higher due to the difference in handling zero recombination values) were located within 10 kbp of the significant slopes and 65 falls in the top 0.1 percentile for the H12 statistic (Fig. 4B). The SNP markers surrounding candidate genes of CO3-17 and LS2-17 were significantly (*p *< 0.05) enriched in the top 0.1 percentile in both selection scans (Table S9) using hypergeometric distribution tests. 710 and 685 genes fell within 10 kbp of the top peaks, yielding 13 and 15 GO-term enrichments for iHS and H12 selection estimates respectively (Fig. 5, Table S8).

### Autumn phenology

One hundred sixty four and six significant slopes were observed for field and phytotron autumn phenology traits and the extended slopes contained 336 and 30 candidate genes, respectively. Of the 366 candidate genes, 34 were shared between different fall phenology traits and 6 were shared for the same trait measured in different years in the field. We observed overlaps between years for CO3 and LS2 only, but no overlaps were seen for CO8 and LS5 (Fig. S6, Table S10). Twenty three candidate genes were shared between two different autumn phenology traits and five candidate genes were shared between four autumn phenology traits in the field. The five candidate genes were shared between CO3-17, CO3-18, LS2-17 and LS5-18 (Table S10).

**Fig. 6:**
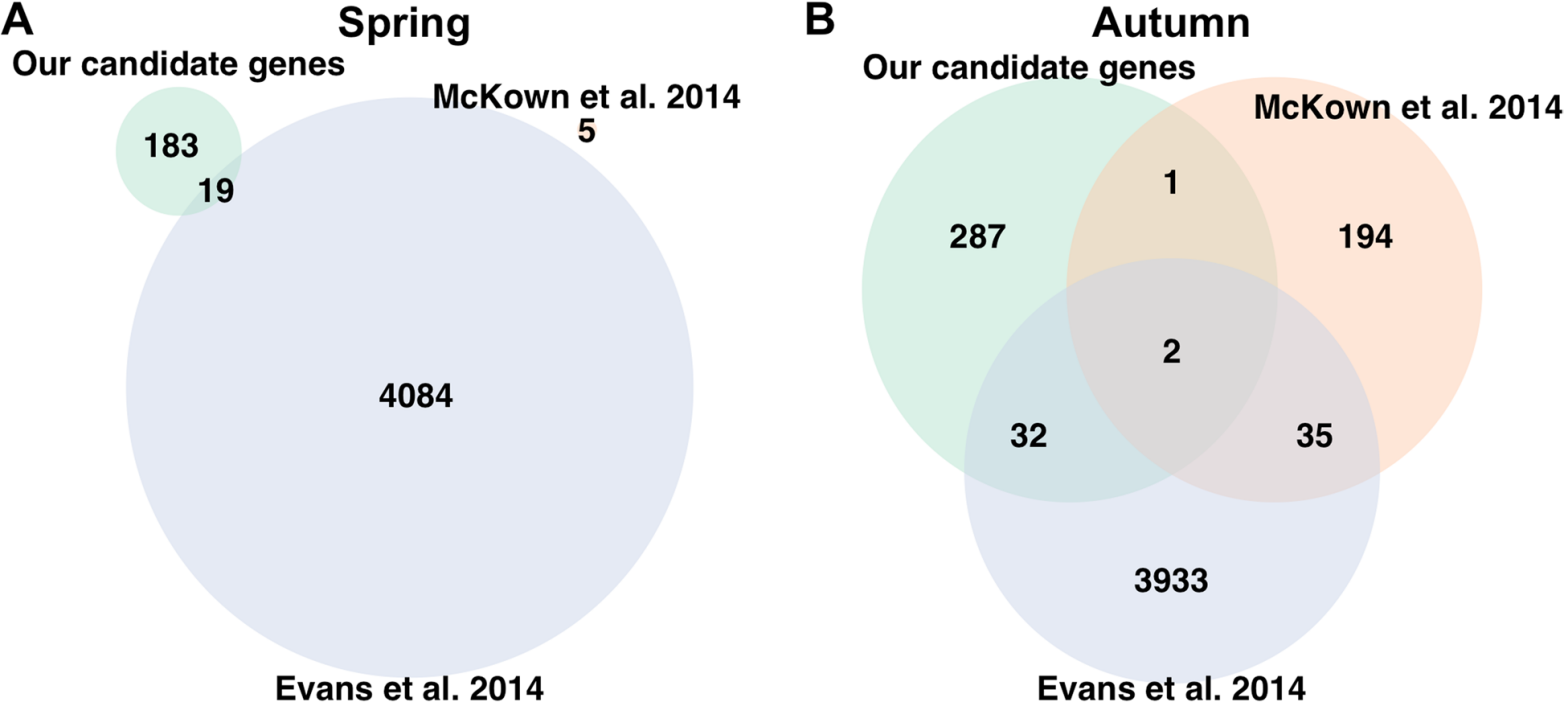
Putative candidate genes in introduced population and native North American range. Overlap between our **(a)** autumn phenology and **(b)** spring phenology (bud burst) candidate genes and those identified in two other studies (Evans [22] and McKown [16])

Two candidate genes for autumn phenology traits were shared between our study and Evans [23] and McKown [16]. Thirty two candidate genes were shared between Evans [23] and our results and one candidate gene was shared between McKown [16] and our results (Fig. 6A, Table S11). The AT GO-term enrichment analysis yielded 13 enrichments for autumn phenology (Fig. 5), 10 of which were found in BS (Fig. 5B). Four of the 13 enrichments were shared with selection estimates, 2 with H12 and 3 with iHS (Fig. 5), of which one was shared between both selection estimates and field autumn phenology traits (Fig. 5A).

### Spring phenology

**Fig. 7:**
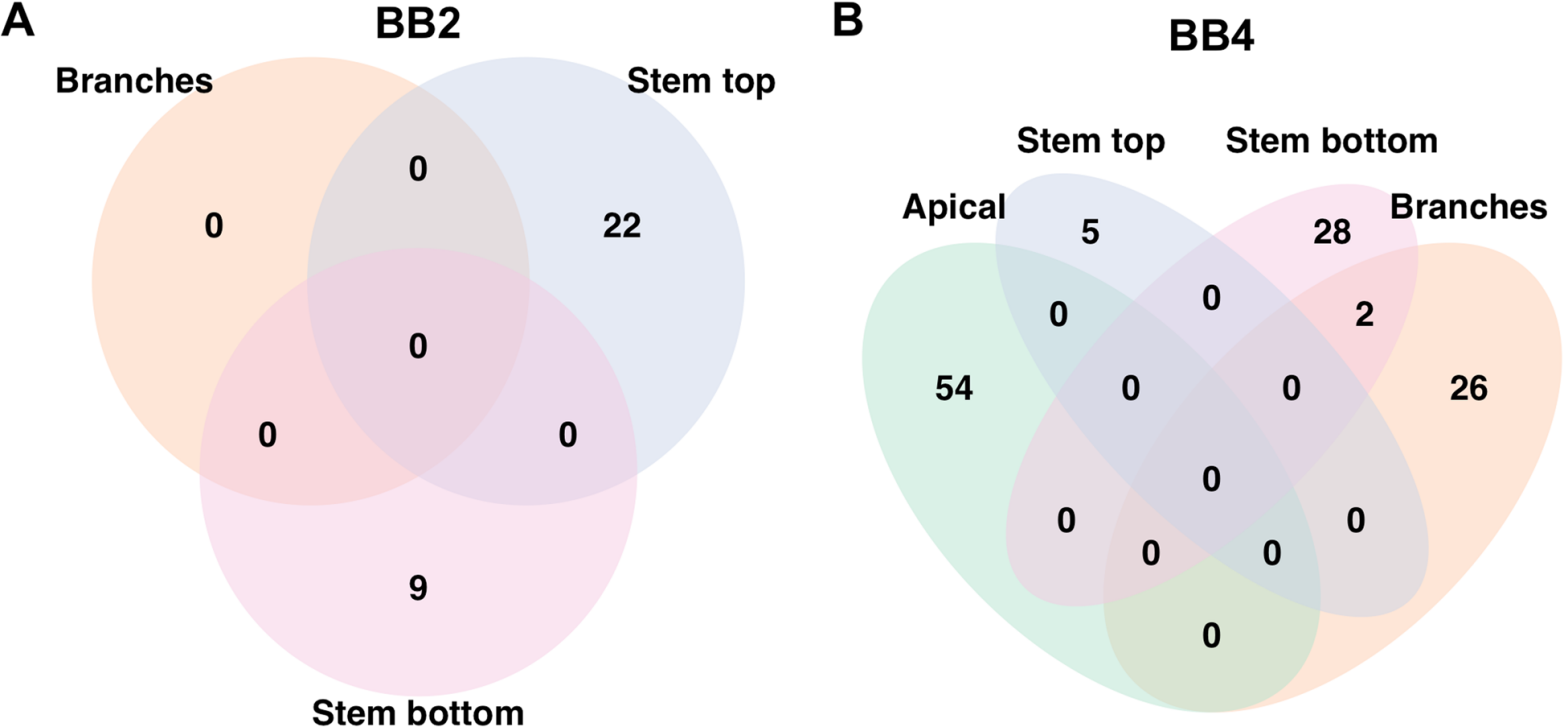
The candidate gene overlap in the four different bud types in phytotron. **a** The initiation stage (BB2) and **(b)** for the completion stage (BB4)

In total 21 and 55 significant slopes were observed for field and phytotron spring phenology traits and the extended slopes encompass 61 and 148 candidate genes, respectively. Five genes were shared between the same trait across years in the field and 2 were shared between BB4-stb and BB4-brn in the phytotron (Fig. S6, Table S10). These 2 candidate genes constitute the only overlap for the phytotron bud burst as the four phytotron bud burst traits showed no within stage overlap in candidate genes for either stage, BB2 or BB4 (Fig. 7). No overlap was observed between the phytotron and field estimates for these stages either (Table S10). Additionally, a total of 7 candidate genes were shared between at least one autumn and one spring phenology trait.

For spring phenology traits, we observed no candidate genes that were shared between our results and McKown [16], while a total of 19 genes were shared between our results and Evans [23] (bud burst) (Fig. 6B, Table S11). There were 28 enrichments found for spring phenology traits in the AT GO-term analysis (Fig. 5). Finally, 2 GO -term enrichments were shared between spring phenology and H12 and iHS selection estimates respectively (Fig. 5).

## Discussion

### Genome wide association study issues

The relatedness structure of our mapping population and the small size of the study population both presented challenges for conducting an unbiased GWAS. Even if the mapping population we have used is small and family structured, the population display similar extents of LD than have previously been reported from samples obtained from wild individuals [24, 25], suggesting that the structure and any previous selection have not had major effects on the level of LD in the population. We also attempted to mitigate the issue of the relatedness of individuals by including a kinship matrix in the GWAS analyses [26, 27], though this is unlikely to remove the confounding effect of relatedness fully. The small size of our study population likely presents another problem for the power of our GWAS. However, this population has previously been found to harbor substantial genetic variability in phenology traits, sufficient for future adaptation [22]. Thus, while much of the smaller effect architecture will likely remain undetected, we should have the power to locate at least large effect loci within the traits [15, 16, 23] despite the small size of our population. Another factor in mitigating both of these issues is our choice to use the Lindley method [28], allowing us to utilize the information contained in the full 7,076,549 set of SNP’s and their respective linkage statuses, though this is unlikely to fully remove false positives caused by the small size, structure and previous selection in this population. However, despite our best efforts to mitigate these issues, we acknowledge our GWAS results are not globally applicable to *Populus trichocarpa* as species, but specific to our study population to further explore the possibility of better adapting it to the novel conditions of Northern Europe for breeding purposes.

### Phenotypic variation and heritability

Despite the size and structure of the population, vast majority of the traits displayed appreciable levels of phenotypic variation (Fig. 1) and had appreciable levels of both narrow and broad sense heritability (Table S7). The clear exceptions to this were BB2-top and BS2 which both showed a general lack of phenotypic variation as well as low narrow sense heritabilites and overfitting in the model used for estimating the BLUP’s used in broad sense heritability estimations. (Fig. 1E & F, Table S7). Heritabilities are known to vary between different environments, though for most species and traits, measurements taken in a controlled environment usually produce higher heritability estimates [29], but our results show the opposite trend. BB2-top and BS2 are normally the traits that best capture the timing of when plants exit and enter dormancy, respectively and their drastic lack of phenotypic variation and heritability are thus slightly concerning. However, this can at least partially explained by the damage to the apical buds sustained by many individuals in our phytotron experiment. As such, we decided to exclude these two traits from further analyses. Finally, overfitting seemed to be a considerable issue in broad sense heritability estimates in the phytotron with BB2-brn, BB2-stt. BB2-top, BB4-brn, BB4-top and both initiation and completion of bud set models for BLUP -estimation overfitting, while of the field traits CO8-18 and LS5-18 were overfit. The generally lower numbers of observations might at least partially explain the overfitting problem, especially in the phytotron.

### Temperature and water availability may have had effect on phenology transitions

We observed drastic differences in temperature and precipitation across the two years 2017 and 2018 in the field trial. Year 2018 was hotter, with monthly mean temperatures consistently at least ~ 2 °C higher from April until August, with more considerable difference (3–5 °C) in temperatures in April, May and July. In 2017 the average temperature was above 5 °C from May to October with an average temperature of 12.8 °C whereas the average temperature in 2018 was above 5 °C already in April and lasted until October with an average temperature of 13.9 °C. The difference in mean temperature is largely caused by an early and very warm spring and an exceptional heatwave during July in 2018. Furthermore, year 2018 had lower levels of precipitation, and the end of the growing season may have been affected by lowered water availability as well (Table S2), though the availability was not directly measured.

Temperature is the main driver of the initiation of spring phenology transitions in many annual and perennial species, including *Populus* [4]. Furthermore, higher temperatures have been seen to lead to faster completion of bud burst in other species [30, 31]. As such, it is no surprise that we observed earlier initiation and completion of spring phenology transitions in 2017 compared to 2018 (Fig. 1). Even though the initiation of autumn phenology is largely driven by day length in *Populus* [2], there is some evidence that both temperature and water availability may affect both the timing of initation and completion of autumn phenology transition, especially in terms of leaf phenology [32–34]. There is also some evidence of earlier spring phenology transition leading to an earlier autumn phenology transition [35]. Similarly, water availability and rain patterns have been seen to affect both initiation and speed of autumn phenology, though the direction of these effects seem species dependent [33]. Our findings are well in line with previously established relationships between temperature, rainfall, water availability and spring phenology. As such, it is likely that the conditions during year 2018 affected the earlier onset and completion of autumn senescence in the year, though it yet remains unclear to us which of these relationships were the causal ones. On top of the phenotypical differences observed, we also identified noticeably fewer significant slopes for autumn phenology traits in 2018 compared to 2017 (Table 1), which could be indicative of differential genetic control of autumn phenology in year 2018, though further work is required to confirm such claims.

### Genetic architecture of phenology and growth

Linkage disequilibrium (LD) decays over, on average, 10 kbp (Fig. S5) in our *P. trichocarpa* population, which in similar to earlier studies in *P. trichocarpa* in its native range [24]. Based on this extent of LD we augmented all significant slopes by 10 kb in either direction in order to identify possible candidate genes. We identified a total 250 significant slopes across the study traits with sufficient genetic variation. Among the traits, LS5-17 has more than three times as many significant slopes than the trait with next highest number of slopes (Fig. 3A, Table 1). This supports earlier evidence for the complex genetic basis of autumn phenology traits in *P. trichocarpa* [16, 23]. For growth (DBH-17) we only identified 4 significant slopes (Fig. 3D, Table 1) likely due to the highly polygenic nature of growth traits and the low power to detect loci with small effects due to the small size of our population and due to, to lesser extent, limitations of even the modern GWAS methods [36, 37].

Changes in lipid and protein metabolism have been previously observed during phenology transitions in *Populus* and other tree species [38, 39]. Lipid contents of various membranes are well-established indicators of cell status, such as cold hardiness [39, 40]. Our GO-term analyses support this as we uncovered 5 enrichments across our traits that are directly linked to membrane structures three of which were in GO-term plasma membrane (GO:0,005,886) found in BB-18, BS and CO-17 (Table S8).

### Signatures of positive selection and GO-term enrichment

Positive selection often drives local adaptation [7], leaving detectable signatures in genetic variation across the genome. Using two test statistics, iHS [41] and H12 [42], we identified clear signatures of positive selection at multiple locations across the genomes of our *P. trichocarpa* study population. We observed hundreds of markers within 10 kbp of significant slopes in the top 0.1 percentile of estimated selection values for both of the selection scans (Fig. 4). Thus, many of the significant slopes we identify likely also correspond to genome regions that have been under positive selection in the native environments of the parents of our study population.

The GO-term analysis of putative candidate genes under selection also yielded enrichments in plasma membrane (GO:0,005,886) for both statistics, chloroplast envelope (GO:0,009,941) for iHS and membrane (GO:0,016,020) for H12, lending further support to the importance of membrane structures for adaptation to northern climates [39, 40]. Among other noteworthy enrichments was response to cold (GO:0,009,409) enriched for iHS (Table S8) intuitively linking to both autumn and spring phenology as some aspects of both have been found to be temperature dependent in *Populus* [2].

### Autumn phenology

Initiation of both bud set and leaf senescence has been previously observed to be consistent between years and conditions for the same trees, suggesting a more stringent genetic control of the initiation of autumn phenology traits [2, 22, 43]. Our results are in line with this, as the 11 candidate genes we found shared between years were exclusively found in the early stages (CO3 and LS2) (Fig. S6, Table S10). Similarly, autumn phenology traits have been previously shown to have a degree of shared genetic architecture [16] and be genetically correlated in this population [22]. Our results support this as we observe a notable overlap in candidate genes between different autumn phenology traits in the field including shared associations of five candidate genes across four field traits. The largest of these overlaps occurring between CO3-18 and LS5-18 and encompassing 15 candidate genes (Table S10), could potentially be taken as further evidence of a systematic stress response to heat and/or drought (Table S10, Table S2). The senescence hastening effects of these stresses have been observed in model species [16, 44] fitting well with our observations in 2018. The overlap we observe may in part have been driven by false positives, possibly due to earlier selection in the population for growth and adaptation. However, even if the latter is true, it does not alter the fact that same genes have been under artificial selection in the traits and as such are likely important for adaptation to the novel conditions.

We identified two candidate genes for autumn phenology traits that were also identified in the earlier studies of Evans [23] and McKown [16] (Fig. 6A). These genes were glucan synthase-like 12 (Potri.003G214200) shared between LS2-18 and bud set in both Evans [23] and McKown [16], and additional traits such as yellowing and leaf drop in the latter, and glucuronidase 2 (Potri.015G049100) shared between LS5-17 and leaf drop in McKown [16] and bud set in Evans [23]. As both of these have functions in metabolism of complex carbohydrates (https://uniprot.org), they may have roles in cell wall degradation or production of storage carbohydrates. An additional 34 candidate genes that we identify in this study are also shared with Evans [23] but only a single gene is shared with McKown [16] (Fig. 6A). These overlaps are similar in magnitude to previous comparisons [45] and suggest that the some of the larger effect loci of genetic architecture of autumn phenology traits are shared across both native and novel environments, and observable even in our small and structured population. However, the relatively large numbers of study specific candidate genes could also hint at the complexity of these traits under the variable natural conditions. The lack of overlap between genes shared between the two years in our results under the novel conditions of northern Sweden and the genes found in the two studies across the native range [16, 23] could indicate that there are novel stresses in the non-native conditions, highlighting the need for unique allele combinations needed for optimal adaptation.

Both the selection statistics showed significant enrichment with GWAS hits in the top 0.1 percentile for CO3-17 and LS2-17 (Table S9). This suggests that the start of the autumn phenology transition is a more important adaptation than the completion, a view that has been supported in earlier studies in *Populus* [2]. Furthermore, the fact that we observe significant enrichments in these two traits but fail to detect any enrichments in the corresponding traits in 2018 lends support to our view that different environmental factors were driving autumn senescence across the two years. These observations suggest that results from the more usual year of 2017 and in particular the candidate genes we identify, are more relevant for driving local adaptation.

### Spring phenology

The apical meristem has more functions than axillary meristems in species with apical dominance. One such extra function is the inhibition of axillary buds through apically produced auxins [46, 47] that are basipetally transported to the buds along the stem [48]. We studied the timing of bud burst of different buds in our phytotron experiment and the results reflect the apical dominance effect, as the apical bud (top) initiates bud burst earlier than other types of buds (Fig. 2A). Furthermore, the stem top 50% (stt) and stem bottom 50% (stb) buds display considerably different timing of bud burst conditional on the presence of a functional apical bud (Fig. 2B & C). As such, our findings are in line with the previous reports of suppression of other buds by apical bud [46, 47]. Our findings underline the importance of the apical bud for repressing bud burst of stem buds during spring phenology transitions. Branch buds, however, seemed to operate independently from the apical bud, with no significant difference between undamaged and damaged apical buds (Fig. 2D).

The higher numbers of significant genes observed in the phytotron in combination with a general lack of overlap in candidate genes between bud burst traits within our study could support not only the well-established view that bud burst is a highly plastic trait but also that is has a complex genetic basis [16, 23]. The lack of overlap in candidate genes identified between the bud burst traits in the phytotron experiment suggests that the genes controlling bud burst in different parts of the plant are unique (Fig. 7), which may have an effect on the comparability of results between studies. While these results may seem striking, it is worth keeping in mind that our small and structured population required the use of stringent thresholds for Lindley score and less strongly associated associations for bud burst traits could therefore have been filtered out and would represent false negatives. Nonetheless, our results show that no large effect loci are shared between bud burst in different parts of the plant or between years.

A total of 5 candidate genes were identified for bud burst, with 2 and 3 shared between the initiation stage BB2 and the completion stage BB4 respectively. This aligns nicely with the genetic correlations found previously [22], but somewhat surprisingly suggests a more consistent genetic control in bud burst between years than in autumn phenology (Fig. S6). Comparing our results to Evans [23], we observe an overlap of 20 genes candidate genes, suggesting that there is a degree of similarity in the genetic control of bud burst between the native and the novel environments. However, this overlap is substantially lower that what we observed for the autumn phenology traits (Fig. 6A & B).

### Summary of phenology candidate genes for adaptation to northern Europe

Candidate genes identified for phenology traits across the two years offer potential targets for adaptive improvement of *P. trichocarpa* to northern European conditions as they are stable across the two very different years, though complementary studies are required to confirm the roles of the specific candidate genes due to the weaknesses of our population. In general, we observed candidate genes with functions in auxin metabolism, lipid metabolism and as helicases (Table S10), each of which have intuitively fitting functions for timing or speed of phenology transitions. Auxins have a well-established role in delaying of senescence in plants [49, 50]. The timing of the initiation of senescence has been previously observed to be under stringent genetic control in *Populus* [2, 22, 43], suggesting that the initiation rather than completion of autumn senescence is of greater importance for adaptation. Lipid contents of various membranes are indicators of cell status, such as cold hardiness [39, 40], which is a key part of winter dormancy. Spring and autumn phenology transitions represent larges transcriptomic changes in the yearly life of perennial plants at northern latitudes [51]. Many of the changes that perennial plants exhibit during these transitions are also dependent on prerequisite conditions being met. For example, in *Populus* bud set has been observed to be a prerequisite for leaf shed [2]. As the summer of 2018 was extremely warm, the high number of genes shared between CO and LS could offer insight into stress induced senescence. The genes shared between the traits would seem to be in agreement with previously established roles of the cell wall and cytokines in phenology transitions [50, 51].

## Conclusions

The study presented here is the first to study the genetic basis of phenology traits in a population of *P. trichocarpa* introduced to northern Europe. We find considerable and heritable phenotypic variation in and complex genetic architectures underlying most of the studied phenology and growth traits, and identify multiple putative candidate genes despite the small and structured study population. Many of the candidate genes we identify function in cell membranes or cell wall, which both have significant biological functions during phenology transitions in the novel environment of northern Europe. Comparison of candidate genes with studies performed in the native range show some overlap for both autumn and spring phenology transitions although the latter show far less overlap. Aside from these observations we find evidence for significant enrichment of SNPs under selection in significant slopes for the initiation steps of autumn phenology transitions. These findings are in line with earlier observations of a more genetically well-defined control of the initiation of autumn phenology compared to both the completion of autumn phenology transitions and spring phenology in general. Together with results from Richards [22] in the same population it strongly seems there is potential for adaptive improvement in our small *P. trichocarpa* population, though these results are likely highly population specific and any application of the results presented here to other populations of *P. trichocarpa* should be done carefully. Our findings here will hopefully encourage smaller scale tree breeders, showing that massive collections are not necessarily needed for adaptation to novel conditions in highly outcrossed species.

## Methods

### Plant material, phenotyping of field experiment and climate data

The trees used in this study are first- or second-generation offspring generated from crosses between *P. trichocarpa* trees collected from across the natural range in western North America (Table S1). Individuals with high growth and well-adapted phenology timing were previously chosen from 34 families with 1 to 21 full-sibs per family utilizing a screening trial. Chosen individuals were then clonally replicated and planted in 2003 in five complete blocks together with some of the parent and unrelated non-parent trees at Krusenberg near Uppsala, Sweden (59°44′44.2"N 17°40′31.5"E). At the time of this study (2017–2018), 564 live ramets from 109 unique genotypes remained at the field site, all of which were genotyped with additional 12 non-field individuals. Climate data for the field site was obtained from the SLU Ultuna climate station (Table S2). No permissions were required to sample the plant material.

The trees were phenotyped every 2–5 days in two successive years, 2017 (17) and 2018 (18), for bud burst (BB) (spring phenology) and for leaf shed (LS) and autumn coloring (CO) (autumn phenology). Bud set (BS) is the most relevant measurement of season-ending growth [2], but is very hard to measure accurately in fully grown trees. Leaf shed and autumn coloring were measured as proxies for bud set as they are easy to measure in adult trees and have been observed to happen only after bud set [2]. The diameter at breast height (DBH) of the trees was measured in 2017 and was used as a proxy for lifetime growth.

Bud burst was scored using a scale with six steps, ranging from fully dormant buds [1] to fully opened buds with unfurled leaves and active shoot growth [6] (Table S3). Leaf shed was scored on a scale ranging from 1 to 5, with each stage describing a window of 20% of leaves shed (stage 1: 0% to 20% leaves shed, stage 5: 80% to 100% leaves shed). Autumn coloring was measured slightly differently between the two years, using a scale ranging from 1 to 5 in 2017 and a scale ranging from 1 to 8 in 2018 (Table S4), based on the level of yellowing in the leaf crown with 1 being fully green leaves and 5/8 being fully yellow leaves.

### Plant material, conditions and phenotyping of phytotron experiment

In February of 2018, cuttings were taken from 99 clones in the Krusenberg field trial. These cuttings were taken from stems of root suckers growing from stumps of thinned individuals or from branches of mature trees and stored at—4 °C until planted in pots in March of 2018. Two cuttings of similar length (~ 10 cm) from the same clone were planted in each pot. This was done in three replicates to produce a 3-block randomized design. After sprouting, the less vigorous cutting was removed leaving only one cutting per pot. Cuttings were then put through two simulated seasons described in Table S5.

Bud burst and bud set were measured during the second simulated season (Table S5). Bud burst was scored following the six-step scale used in the field trial (Table S3), but the buds on the saplings were divided into four classes and scored separately. These classes were the apical bud on the longest stem (top), the branch buds (brn) consisting of all the buds on lateral branches if present, the top 50% of the stem buds (stt) and the bottom 50% of the stem buds (stb) consisting of all the buds on the top and the bottom half of the main stem respectively. Bud set was scored in the highest situated undamaged bud, apical bud if it remained undamaged, following a seven-stage scale introduced in Rohde [43] with minor changes in stage numbering, ranging from growing apical meristem (1 in our numbering, 3 in Rohde [43]) to fully set bud (7 in our numbering, 0 in Rohde [43]).

### Genotyping, SNP calling and filtering

Leaf samples were collected from trees in the Krusenberg trial in the autumn of 2016 and stored dried with silica gel until DNA extractions. DNA extractions were repeated for a few clones using leaves taken from the cuttings used for the phytotron experiment. Genomic DNA was extracted using a Macherey–Nagel NucleoSpin® Plant II kit according to manufacturer’s instructions. Quality and concentrations of the DNA was assessed using a NanoDrop spectrophotometer. Paired-end sequencing libraries with insert sizes of 350 bp were constructed for all samples at the National Genomics Infrastructure at the Science for Life Laboratory in Stockholm, Sweden. Whole-genome sequencing with a target depth of 20 × was performed using an Illumina HiSeq X platform with 2 × 150-bp paired-end reads, generating on average 47.6 M reads per sample with median depth of 21.6.

Sequencing reads for all accessions were mapped against the reference genome of *P. trichocarpa* v3.0, using BWA-MEM (v0.7.17) [52] using default parameters. Depth and breadth of coverage were assessed in order to confirm that all samples had a minimum coverage of 10X (range from 12 to 49X, see Table S6) Post-mapping filtering removed unmapped reads (samtools v1.10) [53] and tagged duplicate reads (picard MarkDuplicates v2.10.3) (http://broadinstitute.github.io/picard/), which did not exceed 14% of the libraries (ranging from 3 to 13.8%).

We used GATK v3.8 [54] to call variants. We performed local realignment around indels with RealignerTargetCreator and IndelRealigner (default parameters). Sample variants were called using HaplotypeCaller, producing gVCF files (-ERC GVCF). Samples were hierarchically merged into intermediate gVCF files using CombineGVCFs and were finally called jointly with GenotypeGVCFs. SNPs were selected using SelectVariants and filtered with VariantFiltration (QD < 2.0; FS > 60.0; MQ < 40.0; ReadPosRankSum < -8.0; SOR > 3.0; MQRankSum < -12.5). SNPs were pruning with vcf/bcftools [53, 55] to remove positions with extreme depth (min-meanDP 16, max-meanDP 33; these thresholds correspond to the average depth ± one standard deviation), missing in more that 30% of the samples, non-biallelic SNPs with minor allele frequencies < 0.05, or SNPs displaying an excess of heterozygosity (FDR < 0.01). This resulted in a data set consisting of 7,297,862 SNPs. SNPs were further filtered for allele number = 2, minor allele frequency (MAF) > 0.05 and extreme deviation from Hardy–Weinberg equilibrium (HWE) < 10^–6^. After filtering, 7,076,549 SNPs were retained and used in all downstream analyses.

### Missing data and phenotypic data imputation

During the field experiment individual trees sometimes passed through more than one phenology stage between two successive phenotypings. To account for these missing phenotypes, we first converted each ordinal stage into the number of Julian days (measured from January 1) when a stage was first observed for a given individual tree. This was done to remove accidental reversals of phenology stages which occurred at low frequencies in the data set due differences in subjective scoring by different observers or through shedding of yellowed leaves which sometimes case an apparent ‘greening’ of some trees. Once an individual tree had transitioned to the next phenology stage the Julian date was recorded for that stage and any subsequent reversals were discarded. For the first (1) stage for both spring and autumn phenology we used the last observed date as the observation point, as the first stage denotes ‘no change’ making earlier observations of the stage uninformative about the progress of phenology.

A local regression model (LOESS) was fitted through the transition days to estimate the missing stage transition days for each individual separately. The method fits a non-linear curve that is not constrained to fit any a-priori distribution for each individual separately. This allows estimation of the day in which these individuals entered each ordinal developmental stage allowing us to include individuals not observed at transitions between stages in the downstream analyses (For more information see Richards [22]). The mean of each clone was then calculated and used as the stage-specific phenotype for the genotype in all subsequent analyses. We observed negligible differences between estimated BLUPs and means (Fig. S1) due to the simplicity of our experimental design.

### Choice of stages

The phenology traits were phenotyped using multiple stages that are highly correlated across individuals (Fig. S2). Most information is conferred by the initial transition (stage 1 to 2) and final transition stages in terms of growth period and vulnerability to damage and we therefore chose these two stages to serve as representative time points for phenology transitions in our data. For bud set and leaf shed, the stages used were the second and the last stage. For autumn coloring, the last stage was chosen to represent the end of the phenology transition, but here we instead used the third stage to represent the initiation stage, as the third stage was directly phenotyped in both years (Table S4). For bud burst, the start of the second stage was chosen as the beginning of phenology transition, and the fourth stage was chosen as the end of transition as this represents the stage when leaves begin to emerge and unfurl beginning the active photosynthesis.

For the field phenology traits, a Welch two sample t-test was performed to confirm whether the differences observed in phenology timing between the two years was statistically significant. Broad (H^2^) and narrow sense heritabilities (h^2^) were also calculated for each of our chosen traits using the R packages (R Core Team, 2014) “inti”, utilizing the Cullis -method [56] using a simple model for estimating BLUP’s, where the repeat and status of apical bud (if applicable) are fixed effects and repeat within block and genotype were random effects, and “heritability”, a marker-based method developed specifically for plant data [57], utilizing the standardized kinship matrix calculated in GEMMA [27] and apical bud status (if applicable) as covariate, using the imputed phenotypes from each individual as replicates for each clone to estimate to what extent phenotypic variation was heritable (Table S7).

### GWAS and Lindley score

Though small and heavily structured, the study population has been previously found to harbor enough genetic variability in phenology traits for adaptation [22]. Thus, we performed a genome wide association study for each of the 23 chosen traits utilizing a univariate linear mixed model implemented in with GEMMA (v. 0.98.1). Due to the aforementioned relatedness structure in our data, consisting of a mixture of individuals spanning the range from full-sibs to unrelated, we utilized a kinship matrix (produced in GEMMA) to partially mitigate the issues caused by the confounding effect introduced by the relatedness structure. All field traits were run with no covariates, but for traits measured in the phytotron, a binary covariate was included to indicate the status of the apical meristem (damaged/not damaged) to account for effects of the phytotron issues (see Results). To take advantage of the large number of markers available, better utilize the information contained in the linkage disequilibrium among adjacent markers and further mitigate the effects of our small and related population, we used the Lindley score-based method introduced by Bonhomme [28]. The local Lindley score is calculated using information derived from multiple adjacent SNPs, thereby limiting the number of tests performed while utilizing all the available data. Each *p*-value that exceeds a user set threshold (ξ, on a logarithmic scale) will contribute positively to a local Lindley score and vice versa for SNPs that fall below the threshold. Lindley scores can not to go below zero regardless of how many p-values that fall below a given threshold. If enough adjacent tests are significant or a single test is highly significant, the local Lindley score will rise above the chromosome specific significance level, signifying an area of interest, which is especially useful for highlighting areas containing multiple weakly significant markers. This threshold is determined for each linked, autocorrelating set of SNPs separately and is essentially the null distribution of said set of markers [28]. The Lindley score is the result of a directional process and the leading-edge slope (hereafter slope) is the area of interest.

### LD decay, candidate genes and candidate gene comparisons

To identify candidate genes around the significant slopes revealed by the local Lindley score analyses, the rate of decay of linkage disequilibrium (LD) was estimated following method of Wang [25]. Briefly, SNP markers were randomly thinned down to 100,000 markers using PLINK 1.9 [58] and remaining markers were used to calculate the squared correlation coefficients (r^2^) between all SNP pairs in non-overlapping 50 kbp windows using PLINK 1.9. The decay of LD across physical distance was then estimated using nonlinear regression of pairwise *r*^2^ against physical distance between sites in base pairs [59]. LD decays over, on average, 10 kbp (Fig. S5) in our *P. trichocarpa* population, and we used this information to determine putative candidate genes for the regions that showed significant association in the GWAS. Boundaries of significant slopes were extended by ± 10 kbp to search for genes using the *P. trichocarpa* v3.1 annotation [60] available at Phytozome 12 (https://phytozome.jgi.doe.gov/pz/portal.html).

The found candidate genes were compared between the years for the field traits and between the different bud set traits for the phytotron experiment. The results of these comparisons were illustrated using Venn diagrams. We compared candidate genes identified for spring and autumn phenology with candidate genes identified in two earlier studies in *P. trichocarpa*, Evans (results from all sites and tests compiled) [23] and McKown [16], to reveal any similarities in candidate genes identified based on common garden data from Sweden, Canada or the phytotron experiment.

GO-term enrichment analysis was performed on each of the study traits by merging the candidate genes from each stage of the trait. The analysis was performed online at PopGenIE (https://popgenie.org) and was performed on the *Arabidopsis thaliana* synonyms of the genes using the default settings of the tool. PopGenIE uses Fisher’s exact test with False Discovery Rate (FDR) correction with a corrected p-value threshold of 0.05 and minimum of two genes by default.

### Signatures of positive selection

We calculated two haplotype-based test statistics to detect positive selection, the integrated haplotype score iHS, [41] and H12, which measures haplotype homozygosity and is especially useful for finding soft selective sweeps [42]. Both test statistics were calculated using selscan v1.2.0a [61]. The genetic map positions of all SNP markers were calculated based on the population averaged recombination rates estimated using LDhat [25] with missing values set to zero. iHS does not produce estimates for zero values leading to slightly different numbers of estimates between the two methods. For both statistics the top 0.1 percentile was used as a threshold for signatures of selection. To test for possible enrichments between SNPs showing evidence for positive selection and significant SNPs from the GWAS, we used hypergeometric distribution tests on each trait separately. Peak SNP’s were then pinpointed for each selection scan using the ggpmisc package (Aphalo, 2020) in R. A peak was the SNP with highest selection scan score of all SNP’s within a window of 20,001 bp centered at that SNP. Only the peaks in the top 0.1 percentile (hereafter top peaks) were used for downstream analyses for both estimates. Genes within 10 kbp of the top peaks were then identified and compared with our GWAS candidate genes. Genes surrounding the selection peaks were analyzed for GO-enrichments using the default settings on PopGenIE enrichment analysis tool.

## Supplementary Information

Below is the link to the electronic supplementary material.

**Additional file1**: **Table S1**: Schematic of crosses and numbers of individuals produced by a cross between the parent individuals. Mothers are on the left (yellow) and fathers on top (blue). The parents in bold and all offspring are present in the Krusenberg field trial. **Table S2**: Monthly mean temperature and rainfall information for years 2017 and 2018. **Table S3**: Drawings, pictures and descriptions of the 6 stages of bud burst. The table is reproduced as given to the field scorers with drawing of stage 3 and pictures of stages 5 and 6 missing. Drawings reproduced on permission of the owner Alfas Pliura of Lithuanian Research Centre for Agriculture and Forestry. **Table S4**: Autumn coloration scoring scheme for 2017-2018. Original 2017 and 2018 describe the original scoring systems used in the corresponding years. converted 2017 shows, which stages the original 2017 scores correspond in the 2018 scoring system. **Table S5**: Phytotron simulated seasons temperature, light conditions and humidity information. The simulated winter shaded in gray. During late July and early August of 2018, a breakdown of the climate chambers occurred causing the simulated winter (marked with *) to be interrupted by high temperatures before resumption of simulated winter temperatures. **Table S6**: Sequencing breadth and depth coverage of the 121 sequenced individuals. Values have been rounded to three decimals. Genotyped parent and non-parent non-offspring individuals shaded with gray. **Table S7**: Heritabilites of chosen traits. Broad sense heritability is calculated using the Cullis method. * evident overfitting. **Table S8**: GO -term enrichment analysis results. **Table S9**: Hypergeometric test results. Results are rounded to 3 decimals. **Table S10**: All candidate genes identified within 10 kbp of significant slopes. **Table S11**: Shared candidate genes between our results and Evans et al. (2014) and McKown et al. (2014). **Figure S1**: Pearson’s correlation between BLUP and mean values of A) bud burst initation in year 2017 (BB2-17), B) autumn coloring initiation in year 2018 (CO3-18), C) bud set completion (BS7) and D) diameter at breast height (DBH-17). * *p *< 2.2-16. **Figure S2**: Spearman’s correlation matrix and heatmap of all our chosen 23 traits. **Figure S3**: Logarithmic *p-*value Manhattan plot. **Figure S4**: Logarithmic *p-*value QQ-plot. **Figure S5**: Level of LD as function of distance. **Figure S6**: Venn diagram of candidate genes for both year for A) bud burst initiation (BB2), B) bud burs completion (BB4) C) autumn coloring initiation (CO3), D) autumn coloring completion (CO8) E) leaf shed initiation (LS2) and F) leaf shed completion (LS5).

## Data Availability

The raw sequencing reads for all samples available from the European Nucleotide Archive (ENA) under study number PRJEB38910 (https://www.ebi.ac.uk/ena/browser/view/PRJEB38910). Breeding values for all phenotypic traits used in the analyses are available from zenodo.org under DOI number 10.5281/zenodo.4059259 (http://dx.doi.org/10.5281/zenodo.4059259). Additional scripts and files used for the analyses are available at https://github.com/parkingvarsson/CLAP.

## Abbreviations

HWE: Hardy–Weinberg equilibrium
GWAS: Genome wide association study
MAF: Minor allele frequency
LD: Linkage disequilibrium
LOESS: Local regression model
FDR: False discovery rate
BB: Bud burst
LS: Leaf shed
CO: Autumn coloring
BS: Bud set
DBH: Diameter at breast height
(2) or (3): Initiation stage of trait
(4), (5), (7) or (8): Finishing stage of trait
(17): Year 2017
(18): Year 2018
brn: Branches
stb: Stem bottom
stt: Stem top
top: Apical meristem on the longest stem
