# Supplementary material for: The genetic basis of adaptation in phenology in an introduced population of Black Cottonwood (Populus trichocarpa, Torr. & Gray)
Source: BMC Plant Biol. 2021 Jul 2;21:317. doi: 10.1186/s12870-021-03103-5 (PMC8252265; doi:10.1186/s12870-021-03103-5)
Supplement: Supplementary file 1 — Additional file1: Table S1: Schematic of crosses and numbers of individuals produced by a cross between the parent individuals. Mothers are on the left (yellow) and fathers on top (blue). The parents in bold and all offspring are present in the Krusenberg field trial. Table S2: Monthly mean temperature and rainfall information for years 2017 and 2018. Table S3: Drawings, pictures and descriptions of the 6 stages of bud burst. The table is reproduced as given to the field scorers with drawing of stage 3 and pictures of stages 5 and 6 missing. Drawings reproduced on permission of the owner Alfas Pliura of Lithuanian Research Centre for Agriculture and Forestry. Table S4: Autumn coloration scoring scheme for 2017-2018. Original 2017 and 2018 describe the original scoring systems used in the corresponding years. converted 2017 shows, which stages the original 2017 scores correspond in the 2018 scoring system. Table S5: Phytotron simulated seasons temperature, light conditions and humidity information. The simulated winter shaded in gray. During late July and early August of 2018, a breakdown of the climate chambers occurred causing the simulated winter (marked with *) to be interrupted by high temperatures before resumption of simulated winter temperatures. Table S6: Sequencing breadth and depth coverage of the 121 sequenced individuals. Values have been rounded to three decimals. Genotyped parent and non-parent non-offspring individuals shaded with gray. Table S7: Heritabilites of chosen traits. Broad sense heritability is calculated using the Cullis method. * evident overfitting. Table S8: GO -term enrichment analysis results. Table S9: Hypergeometric test results. Results are rounded to 3 decimals. Table S10: All candidate genes identified within 10 kbp of significant slopes. Table S11: Shared candidate genes between our results and Evans et al. (2014) and McKown et al. (2014). Figure S1: Pearson’s correlation between BLUP and mean values of A) bud burst initation in [file 12870_2021_3103_MOESM1_ESM.pdf]

The genetic basis of adaptation in phenology in an introduced population of Black cottonwood (*Populus trichocarpa*, Torr. & Gray)

Rami-Petteri Apuli[1], Thomas Richards[1,2], Martha Rendón-Anaya[1], Almir Karacic[3], Ann-Christin Rönnberg-Wästljung[1], Pär K. Ingvarsson[1,*]

[1] Linnean Centre for Plant Biology, Department of Plant Biology, Uppsala BioCenter, Swedish University of Agricultural Science, Uppsala, Sweden

[2] Plant Ecology and Evolution, Department of Ecology and Genetics, Evolutionary Biology Centre, Uppsala University, Uppsala, Sweden

[3] Institute for Crop Production Ecology, Swedish University of Agricultural Science, Uppsala, Sweden

* Correspondence:
Pär K. Ingvarsson, par.ingvarsson@slu.se

Table S1: Schematic of crosses and numbers of individuals produced by a cross between the parent individuals. Mothers are on the left (yellow) and fathers on top (blue). The parents in bold and all offspring are present in the Krusenberg field trial.

		Region	Alaska		British Columbia	Idaho	Oregon	Washington x Oregon	Washington	Open Pollination, Koster, Holland		
		Latitude	58,30 (15)	60,00	49,15	48,25	45,35		45,50 (75)			
Region	Latitude	Clone	**293**	**278**	**193**	214	159	236	**299**	187	209	290
Alaska	58,30	**216**			5		1		5			5
	59,20 (500)	**291**						2		1		
	60,37 (400)	**265**	3	3		1			2	2		
British Columbia	49,00	**230**	7	4		3		2	6			
		Spirit							1			
Idaho x Oregon		237	2	1		3			3	1		3
		**240**		4				1	21		2	
Oregon	44,30	271	1	2				1	4			1
		301								2		1

Table S2: Monthly mean temperature and rainfall information for years 2017 and 2018.

	2017		2018	
	Temp. (^oC)	Rainfall (mm)	Temp. (^oC)	Rainfall (mm)
Jan	-1.6	15.8	-1.4	53.6
Feb	-1.14	19.2	-4.56	19.1
Mar	2.25	28.5	-3.11	23.1
Apr	3.76	26.2	6.05	36.3
May	10.45	10.4	15.33	6.7
Jun	14.7	50.6	16.3	20.7
Jul	16.62	14.6	21.6	81.7
Aug	15.78	61.1	17.77	68.7
Sep	12.16	68.2	12.86	42.2
Oct	6.79	89.9	7.48	21.1
Nov	2.61	70.5	3.41	24.3
Dec	0.18	49.9	-0.28	31.1

Table S3: Drawings, pictures and descriptions of the 6 stages of bud burst. The table is reproduced as given to the field scorers with drawing of stage 3 and pictures of stages 5 and 6 missing. Drawings reproduced on permission of the owner Alfas Pliura of Lithuanian Research Centre for Agriculture and Forestry.

Drawing of stage	**Picture of stage**	**Description of stage**
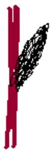	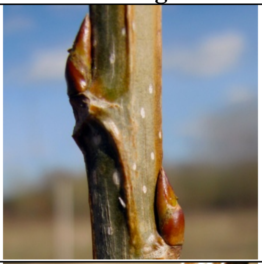	**Stage 1**. Bud swollen, no initials visible. Stage 1,5 if ca 1 mm initial tip visible while bud scales are completely closed
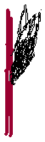	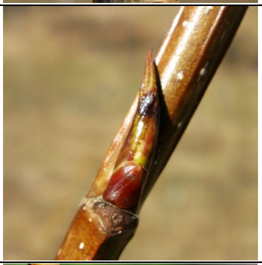	**Stage 2**. Buds are opening, initials visible protruding up to one length of bad scales.
NA	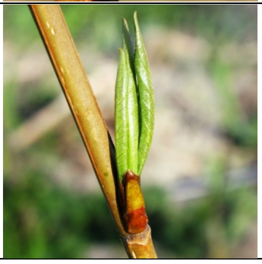	**Stage 3**. Buds are still opening, the leaf primordia elongated, rolled up and longer than bud scales

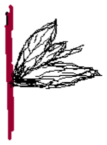	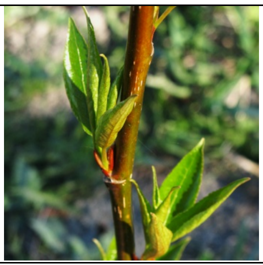	**Stage 4.** Leaves half-shed, bad scales falling off. Stage 4,5 if most of the leaves but not all are shed.
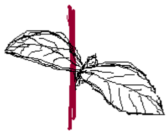	NA	**Stage 5.** Leaves completely shed
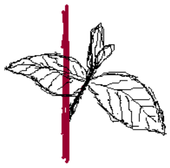	NA	**Stage 6.** >1 cm shoot increment, leaves completely shed

Table S4: Autumn coloration scoring scheme for 2017-2018. Original 2017 and 2018 describe the original scoring systems used in the corresponding years. converted 2017 shows, which stages the original 2017 scores correspond in the 2018 scoring system.

Percentage of yellowing	Original 2017	Original 2018	Converted 2017
0%	1	1	1
		2	
25%	2	3	3
		4	
50%	3	5	5
75%	4	6	6.5
		7	
100%	5	8	8

Table S5: Phytotron simulated seasons temperature, light conditions and humidity information. The simulated winter shaded in gray. During late July and early August of 2018, a breakdown of the climate chambers occurred causing the simulated winter (marked with *) to be interrupted by high temperatures before resumption of simulated winter temperatures.

Starting date	Season	Temperature day (°C)	Temperature night (°C)	Day length (h)	Light intensity (umol)	Humidity (%)
20.03.2018	1	22	18	18	350	80
24.04.2018	1	22	18	8	100	80
01.05.2018*	1	4	4	8	100	90
10.09.2018	2	10	10	12		
20.09.2018	2	15	15	15		
30.09.2018	2	17	17	18		
10.10.2018	2	16	16	17		

20.10.2018	2	11	11	15		
30.10.2018	2	7.5	7.5	12		
09.11.2018	2	4	4	10		
19.11.2018	2	4	4	7		
29.11.2018	2	4	4	6		
09.12.2018	2	4	4	7		
19.12.2018	2	4	4	10		

Table S6: Sequencing breadth and depth coverage of the 121 sequenced individuals. Values have been rounded to three decimals. Genotyped parent and non-parent non-offspring individuals shaded with gray.

SAMPLE	DEPTH	BREADTH
P11086_101	19.281	0.897
P11086_102	19.59	0.902
P11086_103	19.167	0.899
P11086_104	15.197	0.894
P11086_105	16.726	0.895
P7512_101	30.848	0.911
P7512_102	22.041	0.907
P7512_103	25.771	0.907
P7512_104	19.793	0.906
P7512_105	20.279	0.903
P7512_106	24.214	0.896
P7512_107	27.784	0.908
P7512_109	21.203	0.902
P7512_110	12.205	0.884
P7512_111	20.394	0.906
P7512_112	22.627	0.9
P7512_113	29.538	0.907
P7512_114	24.842	0.911
P7512_115	25.263	0.907
P7512_116	18.111	0.906
P7512_117	20.471	0.9
P7512_118	24.39	0.904
P7512_119	12.887	0.894
P7512_120	25.466	0.906
P7512_121	30.1	0.906
P7512_122	18.896	0.903
P7512_123	22.537	0.906
P7512_124	12.746	0.895
P7512_125	19.917	0.901
P7512_126	19.027	0.902
P7512_127	23.981	0.908
P7512_128	17.109	0.899
P7512_129	17.024	0.902

P7512_131	18.485	0.901
P7512_132	21.5	0.904
P7512_133	13.617	0.898
P7512_134	12.272	0.895
P7512_135	21.195	0.903
P7512_136	19.323	0.902
P7512_138	23.152	0.907
P7512_139	17.866	0.902
P7512_140	15.47	0.898
P7512_141	21.393	0.906
P7512_142	24.329	0.904
P7512_143	18.81	0.903
P7512_144	19.342	0.9
P7512_145	25.089	0.909
P7512_146	44.43	0.918
P7512_147	23.154	0.903
P7512_148	21.279	0.909
P7512_149	20.692	0.908
P7512_150	19.929	0.905
P7512_151	21.089	0.904
P7512_152	20.233	0.903
P7512_153	21.629	0.906
P7512_154	26.59	0.908
P7512_155	25.857	0.909
P7512_156	23.673	0.908
P7512_157	28.413	0.91
P7512_158	24.345	0.912
P7512_159	17.221	0.902
P7512_160	18.091	0.902
P7512_161	17.535	0.903
P7512_162	29.995	0.917
P7512_163	36.829	0.917
P7512_164	26.973	0.912
P7512_165	31.253	0.912
P7512_166	18.89	0.905
P7512_168	14.342	0.899
P7512_169	21.486	0.904
P7512_170	47.833	0.917
P7512_171	18.886	0.903
P7512_172	21.603	0.907
P7512_173	21.431	0.908
P7512_174	38.077	0.917

P7512_175	23.981	0.909
P7512_176	21.224	0.907
P7512_177	30.228	0.919
P7512_178	25.63	0.919
P7512_179	30.368	0.915
P7512_180	22.934	0.905
P7512_181	15.927	0.897
P7512_182	23.244	0.906
P7512_184	31.17	0.91
P7512_185	32.415	0.911
P7512_186	30.165	0.906
P7512_187	45.819	0.916
P7512_188	37.223	0.912
P7512_189	17.618	0.901
P7512_190	19.131	0.9
P7512_191	28.347	0.906
P7512_192	28.183	0.905
P7512_193	26.48	0.908
P7512_194	46.832	0.915
P7512_195	32.954	0.911
P7512_196	15.131	0.898
P7512_201	30.811	0.911
P7512_202	14.874	0.899
P7512_204	33.022	0.912
P7512_205	13.249	0.898
P7512_206	41.296	0.917
P7512_208	31.745	0.912
P7512_209	15.72	0.898
P7512_210	49.304	0.917
P7512_211	46.784	0.913
P7512_212	48.918	0.922
P7512_213	21.381	0.906
P7512_214	32.925	0.916
P7512_215	15.937	0.894
P7512_216	31.262	0.901
P7512_217	24.773	0.872
P7512_219	17.099	0.85
P7512_220	17.526	0.902
P7512_221	43.977	0.914
P7512_222	21.007	0.907
P7512_223	16.213	0.899
P7512_225	37.838	0.922

P7512_226	17.068	0.897
P7512_227	15.671	0.897
P7512_228	13.816	0.892
P7512_229	23.007	0.909

Table S7: Heritabilites of chosen traits. Broad sense heritability is calculated using the Cullis method. * evident overfitting.

Trait	Broad sense heritability	Narrow sense heritability	Number of genotypes
BB2-17	0.831	0.698	109
BB2-18	0.720	0.567	109
BB2-brn	0.546*	0.260	68
BB2-stb	0.420	0.191	92
BB2-stt	0.739*	0.526	92
BB2-top	0.797*	0.050	82
BB4-17	0.851	0.777	108
BB4-18	0.710	0.529	108
BB4-brn	0.719*	0.526	69
BB4-stb	0.825	0.640	91
BB4-stt	0.880	0.786	89
BB4-top	0.596*	0.404	78
BS2	0.223*	0.129	50
BS7	0.685*	0.628	81
CO3-17	0.756	0.593	104
CO3-18	0.691	0.474	108
CO8-17	0.843	0.839	107
CO8-18	0.896*	0.665	102
DBH-17	0.848	0.745	109
LS2-17	0.698	0.464	108
LS2-18	0.781	0.647	109
LS5-17	0.765	0.609	86
LS5-18	0.931*	0.88	105

Table S8: GO -term enrichment analysis results.

Trait	GO type	**GO id**	**Pval (corrected)**	**Statistics**	**Description**
BB-17	Molecular function	GO:0009055	1.747e-03	3/7 | 502/24493	electron carrier activity
BB-17	Cellular Component	GO:0005783	1.768e-02	2/5 | 354/23421	endoplasmic reticulum
BB-17	Molecular function	GO:0020037	9.081e-04	3/7 | 373/24493	heme binding
BB-17	Molecular function	GO:0005506	1.136e-03	3/7 | 365/24493	iron ion binding
BB-17	Molecular function	GO:0004497	1.133e-03	3/7 | 252/24493	monooxygenase activity
BB-17	Biological process	GO:0055114	3.888e-02	3/7 | 1403/24222	oxidation-reduction process
BB-17	Molecular function	GO:0016705	7.032e-04	3/7 | 271/24493	oxidoreductase activity, acting on paired donors, with incorporation or reduction of molecular oxygen

BB-17	Molecular function	GO:0019825	7.045e-03	2/7 | 232/24493	oxygen binding
BB-18	Biological process	GO:0009926	3.199e-02	3/85 | 42/24222	auxin polar transport
BB-18	Biological process	GO:0042742	1.084e-02	6/85 | 176/24222	defense response to bacterium
BB-18	Cellular Component	GO:0010008	2.553e-03	2/86 | 3/23421	endosome membrane
BB-18	Biological process	GO:0048527	1.261e-02	3/85 | 28/24222	lateral root development
BB-18	Biological process	GO:0009808	1.072e-02	2/85 | 4/24222	lignin metabolic process
BB-18	Cellular Component	GO:0005886	1.881e-03	19/86 | 1929/23421	plasma membrane
BB-brn	Biological process	GO:0048364	2.290e-02	2/17 | 99/24222	root development
BB-brn	Biological process	GO:0006812	2.471e-02	2/17 | 108/24222	cation transport
BB-brn	Biological process	GO:0009611	3.139e-02	2/17 | 143/24222	response to wounding
BB-brn	Biological process	GO:0008152	3.322e-02	4/17 | 1033/24222	metabolic process
BB-brn	Biological process	GO:0006351	3.796e-02	2/17 | 90/24222	transcription, DNA-dependent
BB-brn	Biological process	GO:0009058	4.281e-02	2/17 | 179/24222	biosynthetic process
BB-brn	Cellular Component	GO:0008194	1.637e-03	3/16 | 100/24493	UDP-glycosyltransferase activity
BB-brn	Cellular Component	GO:0016758	5.753e-03	3/16 | 193/24493	transferase activity, transferring hexosyl groups
BB-brn	Cellular Component	GO:0016779	6.519e-03	2/16 | 47/24493	nucleotidyltransferase activity
BB-brn	Cellular Component	GO:0016757	9.046e-03	3/16 | 347/24493	transferase activity, transferring glycosyl groups
BB-stb	Biological process	GO:0040007	1.314e-02	2/17 | 21/24222	Growth
BB-stb	Biological process	GO:0009414	1.995e-02	3/17 | 189/24222	response to water deprivation
BB-stb	Biological process	GO:0042538	2.424e-02	2/17 | 49/24222	hyperosmotic salinity response
BB-stt	Molecular function	GO:0035251	3.488e-02	2/39 | 23/24493	UDP-glucosyltransferase activity
BS	Biological process	GO:0010119	2.671e-03	4/108 | 28/24222	regulation of stomatal movement
BS	Biological process	GO:0010227	1.061e-02	3/108 | 17/24222	floral organ aBScission
BS	Biological process	GO:0009414	1.955e-02	6/108 | 189/24222	response to water deprivation
BS	Biological process	GO:0030007	2.467e-02	2/108 | 5/24222	cellular potassium ion homeostasis
BS	Biological process	GO:0030833	4.108e-02	2/108 | 8/24222	regulation of actin filament polymerization
BS	Biological process	GO:0009416	4.418e-02	5/108 | 157/24222	response to light stimulus
BS	Cellular Component	GO:0005634	5.239e-03	22/98 | 2161/23421	Nucleus
BS	Cellular Component	GO:0005885	1.163e-02	2/98 | 9/23421	Arp2/3 protein complex

BS	Cellular Component	GO:0000326	1.364e-02	2/98 | 7/23421	protein storage vacuole
BS	Cellular Component	GO:0005886	1.459e-02	19/98 | 1929/23421	plasma membrane
CO-17	Cellular Component	GO:0005886	2.346e-02	10/37 | 1929/23421	plasma membrane
H12	Cellular Component	GO:0009570	1.325e-02	11/182 | 396/23421	chloroplast stroma
H12	Cellular Component	GO:0005737	2.879e-02	14/182 | 703/23421	cytoplasm
H12	Cellular Component	GO:0005829	4.151e-02	10/182 | 465/23421	cytosol
H12	Biological process	GO:0009793	2.472e-02	11/196 | 335/24222	embryo development ending in seed dormancy
H12	Biological process	GO:0009558	4.726e-02	2/196 | 5/24222	embryo sac cellularization
H12	Cellular Component	GO:0005615	3.880e-02	2/182 | 9/23421	extracellular space
H12	Cellular Component	GO:0016020	4.860e-02	27/182 | 2001/23421	membrane
H12	Biological process	GO:0032876	4.726e-02	2/196 | 5/24222	negative regulation of DNA endoreduplication
H12	Cellular Component	GO:0005634	3.680e-04	37/182 | 2161/23421	nucleus
H12	Biological process	GO:0009626	2.435e-02	4/196 | 33/24222	plant-type hypersensitive response
H12	Cellular Component	GO:0005886	1.022e-02	30/182 | 1929/23421	plasma membrane
H12	Biological process	GO:0006355	4.714e-02	23/196 | 1388/24222	regulation of transcription, DNA-dependent
H12	Biological process	GO:0010583	2.283e-02	6/196 | 79/24222	response to cyclopentenone
H12	Biological process	GO:0009414	2.051e-02	8/196 | 189/24222	response to water deprivation
H12	Cellular Component	GO:0000151	4.475e-02	4/182 | 79/23421	ubiquitin ligase complex
iHS	Cellular Component	GO:0009941	4.334e-02	10/211 | 409/23421	chloroplast envelope
iHS	Cellular Component	GO:0005737	2.780e-02	15/211 | 703/23421	cytoplasm
iHS	Biological process	GO:0042742	2.967e-03	10/222 | 176/24222	defense response to bacterium
iHS	Cellular Component	GO:0005663	1.257e-02	2/211 | 4/23421	DNA replication factor C complex
iHS	Cellular Component	GO:0005615	3.254e-02	2/211 | 9/23421	extracellular space
iHS	Cellular Component	GO:0005634	1.840e-04	42/211 | 2161/23421	nucleus
iHS	Cellular Component	GO:0009505	3.516e-02	8/211 | 262/23421	plant-type cell wall
iHS	Cellular Component	GO:0005886	3.645e-03	35/211 | 1929/23421	plasma membrane
iHS	Cellular Component	GO:0043078	8.432e-03	2/211 | 3/23421	polar nucleus
iHS	Biological process	GO:0009737	7.040e-03	12/222 | 304/24222	response to aBScisic acid stimulus
iHS	Biological process	GO:0009409	1.574e-02	10/222 | 243/24222	response to cold

iHS	Biological process	GO:0009826	3.895e-02	6/222 | 98/24222	unidimensional cell growth
iHS	Cellular Component	GO:0005773	1.699e-02	13/211 | 523/23421	vacuole
LS-17	Cellular Component	GO:0005618	4.422e-02	5/49 | 424/23421	cell wall
LS-17	Molecular function	GO:0048441	4.642e-02	2/48 | 11/24222	petal development

Table S9: Hypergeometric test results. Results are rounded to 3 decimals.

Trait	p-value 99.9th percentile ihs	FDR p-value 99.9th percentile ihs	p-value 99.9th percentile H12	FDR p-value 99.9th percentile H12
DBH-17	0.200	0.338	1	1
BB2-17	0.448	0.580	0.448	0.580
BB4-17	1	1	0.115	0.169
CO3-17	0.001	0.013	0	0
CO8-17	0.078	0.231	0.747	0.865
LS2-17	0.003	0.031	0	0
LS5-17	1	1	0	0
BB2-18	0.327	0.450	0.085	0.134
BB4-18	0.099	0.231	0.076	0.134
CO3-18	0.042	0.186	0.042	0.112
CO8-18	0.055	0.203	0.046	0.112
LS2-18	0.269	0.394	0.269	0.370
LS5-18	0.017	0.095	0.002	0.011
BB4-brn	0.140	0.275	0.036	0.112
BB2-stb	0.105	0.231	0.085	0.134
BB4-stb	0.017	0.095	0.010	0.037
BB2-stt	0.150	0.275	0.876	0.963
BB4-stt	0.475	0.581	0.476	0.581
BB2-top	0.993	1	0.064	0.134
BB4-top	0.092	0.231	0	0
BS2	1	1	1	1

Table S10: All candidate genes identified within 10 kbp of significant slopes.

Gene	Trait(s)	# of traits	Function in *Arabidopsis*
Potri.001G209400	BB2-17, BB2-18	2	Lung seven transmembrane receptor family protein
Potri.001G209500	BB2-17, BB2-18	2	Acyl transferase/acyl hydrolase/lysophospholipase superfa[illegible] protein
Potri.013G094000	BB2-18	1	S-locus lectin protein kinase family protein
Potri.013G094100	BB2-18	1	AMP-dependent synthetase and ligase family protein
Potri.014G188700	BB2-18	1	zinc ion binding;nucleic acid binding
Potri.014G188850	BB2-18	1	zinc ion binding;nucleic acid binding
Potri.017G148900	BB2-18	1	Tic22-like family protein
Potri.017G149000	BB2-18	1	WRKY DNA-binding protein 27

Potri.017G149100	BB2-18	1	NADH-ubiquinone oxidoreductase-related
Potri.017G149200	BB2-18	1	
Potri.003G009700	BB2-stb	1	Protein of unknown function (DUF803)
Potri.003G009800	BB2-stb	1	cox19 family protein (CHCH motif)
Potri.003G009900	BB2-stb	1	pumilio 6
Potri.010G055700	BB2-stb	1	Ankyrin repeat family protein
Potri.010G055800	BB2-stb	1	lysine histidine transporter 1
Potri.010G055900	BB2-stb	1	DNA polymerase alpha 2
Potri.010G056000	BB2-stb	1	DNA-binding storekeeper protein-related
Potri.010G056100	BB2-stb	1	NAD(P)-binding Rossmann-fold superfamily protein
Potri.013G098100	BB2-stb	1	Disease resistance protein (TIR-NBS-LRR class) family
Potri.009G093100	BB2-stt	1	CYCLIN A3;4
Potri.009G093200	BB2-stt	1	TIP41-like family protein
Potri.009G093300	BB2-stt	1	zinc finger protein 7
Potri.009G093400	BB2-stt	1	
Potri.009G093500	BB2-stt	1	1,2-alpha-L-fucosidases
Potri.009G094500	BB2-stt	1	Plant basic secretory protein (BSP) family protein
Potri.009G094600	BB2-stt	1	Plant basic secretory protein (BSP) family protein
Potri.009G094700	BB2-stt	1	Plant basic secretory protein (BSP) family protein
Potri.009G094800	BB2-stt	1	S-methyl-5-thioribose kinase
Potri.009G094900	BB2-stt	1	lipase 1
Potri.009G095000	BB2-stt	1	
Potri.009G095100	BB2-stt	1	UDP-Glycosyltransferase superfamily protein
Potri.009G095201	BB2-stt	1	UDP-Glycosyltransferase superfamily protein
Potri.009G095700	BB2-stt	1	Pseudouridine synthase family protein
Potri.009G095800	BB2-stt	1	GroES-like zinc-binding alcohol dehydrogenase family pro
Potri.009G098000	BB2-stt	1	PTEN 2
Potri.009G098100	BB2-stt	1	Granulin repeat cysteine protease family protein
Potri.009G098200	BB2-stt	1	don-glucosyltransferase 1
Potri.009G098300	BB2-stt	1	UDP-glucosyl transferase 73B5
Potri.009G098400	BB2-stt	1	UDP-glucosyl transferase 73B5
Potri.012G030900	BB2-stt	1	
Potri.012G031000	BB2-stt	1	
Potri.014G071900	BB4-17	1	AAA-type ATPase family protein
Potri.014G072000	BB4-17	1	cytochrome P450, family 704, subfamily A, polypeptide 2
Potri.014G072100	BB4-17	1	cytochrome P450, family 704, subfamily A, polypeptide 2
Potri.014G072150	BB4-17	1	cytochrome P450, family 704, subfamily A, polypeptide 2
Potri.016G011500	BB4-17	1	
Potri.016G011601	BB4-17	1	Leucine-rich repeat transmembrane protein kinase
Potri.016G019400	BB4-17	1	UDP-Glycosyltransferase superfamily protein
Potri.016G019500	BB4-17	1	RNI-like superfamily protein
Potri.016G019600	BB4-17	1	receptor like protein 21

Potri.016G019700	BB4-17	1	Cysteine proteinases superfamily protein
Potri.016G026366	BB4-17	1	Pentatricopeptide repeat (PPR) superfamily protein
Potri.016G026532	BB4-17	1	
Potri.016G027101	BB4-17	1	GRAS family transcription factor family protein
Potri.016G030800	BB4-17	1	protein kinase family protein / protein phosphatase 2C (PP family protein
Potri.016G030900	BB4-17	1	Cystatin/monellin family protein
Potri.016G031000	BB4-17	1	cytochrome P450, family 98, subfamily A, polypeptide 3
Potri.016G137500	BB4-17	1	VACUOLAR SORTING RECEPTOR 2
Potri.016G137600	BB4-17	1	cytochrome P450, family 71, subfamily A, polypeptide 25
Potri.016G137700	BB4-17	1	Lung seven transmembrane receptor family protein
Potri.016G137800	BB4-17	1	Protein of unknown function, DUF599
Potri.016G030500	BB4-17, BB4-18	2	DNA/RNA helicase protein
Potri.016G030600	BB4-17, BB4-18	2	DNA/RNA helicase protein
Potri.016G030700	BB4-17, BB4-18	2	
Potri.016G007101	BB4-17, BS7	2	
Potri.016G007200	BB4-17, BS7	2	Outward rectifying potassium channel protein
Potri.016G007300	BB4-17, BS7	2	FAR1-related sequence 10
Potri.016G007400	BB4-17, BS7	2	
Potri.016G007500	BB4-17, BS7	2	agenet domain-containing protein / bromo-adjacent homolo (BAH) domain-containing protein
Potri.001G160700	BB4-18	1	zinc finger (C2H2 type) family protein
Potri.001G160800	BB4-18	1	GTP-binding family protein
Potri.002G112700	BB4-18	1	homolog of yeast ergosterol28
Potri.002G112800	BB4-18	1	
Potri.002G112900	BB4-18	1	
Potri.002G113000	BB4-18	1	alpha/beta-Hydrolases superfamily protein
Potri.015G099200	BB4-18	1	WRKY DNA-binding protein 75
Potri.015G099400	BB4-18	1	
Potri.015G099500	BB4-18	1	Heavy metal transport/detoxification superfamily protein
Potri.016G030000	BB4-18	1	Leucine-rich repeat protein kinase family protein
Potri.016G030100	BB4-18	1	syntaxin of plants 51
Potri.016G030200	BB4-18	1	syntaxin of plants 52
Potri.016G030300	BB4-18	1	Ubiquitin carboxyl-terminal hydrolase family protein
Potri.016G030400	BB4-18	1	Transducin/WD40 repeat-like superfamily protein
Potri.019G106700	BB4-18	1	Ankyrin repeat family protein
Potri.019G106800	BB4-18	1	
Potri.019G106900	BB4-18	1	
Potri.019G106950	BB4-18	1	
Potri.001G275800	BB4-brn	1	Calcium-dependent lipid-binding (CaLB domain) family pr
Potri.001G275900	BB4-brn	1	RING/U-box superfamily protein
Potri.001G276000	BB4-brn	1	
Potri.001G276050	BB4-brn	1	

Potri.002G188300	BB4-brn	1	Chaperone DnaJ-domain superfamily protein
Potri.002G188500	BB4-brn	1	
Potri.002G188600	BB4-brn	1	Protein kinase superfamily protein
Potri.005G143700	BB4-brn	1	Protein kinase superfamily protein
Potri.006G132700	BB4-brn	1	Tetratricopeptide repeat (TPR)-like superfamily protein
Potri.006G132800	BB4-brn	1	Zinc finger C-x8-C-x5-C-x3-H type family protein
Potri.006G132850	BB4-brn	1	
Potri.006G132901	BB4-brn	1	
Potri.017G006900	BB4-brn	1	Endonuclease/exonuclease/phosphatase family protein
Potri.017G007000	BB4-brn	1	S-adenosyl-L-methionine-dependent methyltransferases superfamily protein
Potri.017G007100	BB4-brn	1	
Potri.017G007200	BB4-brn	1	Transcription factor IIIC, subunit 5
Potri.017G007300	BB4-brn	1	chloroplast outer envelope protein 37
Potri.017G007400	BB4-brn	1	RNA-binding protein
Potri.018G008100	BB4-brn	1	GDSL-like Lipase/Acylhydrolase superfamily protein
Potri.018G008200	BB4-brn	1	
Potri.018G008300	BB4-brn	1	Protein of unknown function (DUF1262)
Potri.018G008400	BB4-brn	1	exocyst subunit exo70 family protein G1
Potri.018G132700	BB4-brn	1	phosphorylethanolamine cytidylyltransferase 1
Potri.018G132800	BB4-brn	1	F-box family protein
Potri.018G132900	BB4-brn	1	Leucine-rich repeat transmembrane protein kinase
Potri.018G132950	BB4-brn	1	
Potri.005G143400	BB4-brn, BB4-stb	2	Protein kinase superfamily protein
Potri.005G143600	BB4-brn, BB4-stb	2	Protein kinase superfamily protein
Potri.001G205200	BB4-stb	1	Auxin efflux carrier family protein
Potri.005G151800	BB4-stb	1	Di-glucose binding protein with Kinesin motor domain
Potri.005G151900	BB4-stb	1	
Potri.005G152000	BB4-stb	1	
Potri.005G152100	BB4-stb	1	HVA22 homologue C
Potri.005G152200	BB4-stb	1	Sas10/Utp3/C1D family
Potri.006G060800	BB4-stb	1	serine-rich protein-related
Potri.006G060900	BB4-stb	1	Major facilitator superfamily protein
Potri.007G096200	BB4-stb	1	Peroxidase superfamily protein
Potri.007G096300	BB4-stb	1	Ribosomal protein S6e
Potri.007G096400	BB4-stb	1	Protein kinase superfamily protein
Potri.010G016200	BB4-stb	1	Small nuclear RNA activating complex (SNAPc), subunit SNAP43 protein
Potri.010G016300	BB4-stb	1	Raffinose synthase family protein
Potri.010G016400	BB4-stb	1	Raffinose synthase family protein
Potri.010G066500	BB4-stb	1	HAD superfamily, subfamily IIIB acid phosphatase
Potri.010G066600	BB4-stb	1	seed gene 1
Potri.010G066700	BB4-stb	1	Eukaryotic initiation factor 4E protein

Potri.010G066800	BB4-stb	1	
Potri.010G066900	BB4-stb	1	proton gradient regulation 5
Potri.010G067000	BB4-stb	1	Family of unknown function (DUF716)
Potri.010G067100	BB4-stb	1	actin-related protein C2B
Potri.012G132600	BB4-stb	1	AGAMOUS-like 6
Potri.012G132700	BB4-stb	1	pfkB-like carbohydrate kinase family protein
Potri.012G132801	BB4-stb	1	
Potri.014G000100	BB4-stb	1	zinc ion binding;nucleic acid binding
Potri.014G000200	BB4-stb	1	
Potri.019G053500	BB4-stb	1	zinc finger (C2H2 type) family protein
Potri.019G053600	BB4-stb	1	ARM repeat superfamily protein
Potri.014G047300	BB4-stt	1	Leucine carboxyl methyltransferase
Potri.014G047400	BB4-stt	1	Protein of unknown function (DUF630 and DUF632)
Potri.018G082100	BB4-stt	1	Protein of unknown function (DUF502)
Potri.018G082200	BB4-stt	1	
Potri.018G082300	BB4-stt	1	
Potri.002G252700	BB4-top	1	HIT-type Zinc finger family protein
Potri.002G252800	BB4-top	1	phytochrome interacting factor 3-like 5
Potri.002G252900	BB4-top	1	heat shock protein 60
Potri.002G253000	BB4-top	1	response regulator 24
Potri.002G253100	BB4-top	1	response regulator 24
Potri.002G253200	BB4-top	1	CLIP-associated protein
Potri.002G254300	BB4-top	1	myosin XI B
Potri.002G254700	BB4-top	1	Transducin family protein / WD-40 repeat family protein
Potri.002G254800	BB4-top	1	RmlC-like cupins superfamily protein
Potri.002G254900	BB4-top	1	GroES-like zinc-binding dehydrogenase family protein
Potri.002G255000	BB4-top	1	ammonium transporter 1;1
Potri.002G255100	BB4-top	1	ammonium transporter 1;1
Potri.002G255200	BB4-top	1	
Potri.002G255300	BB4-top	1	Tetratricopeptide repeat (TPR)-like superfamily protein
Potri.002G255400	BB4-top	1	RPM1 interacting protein 13
Potri.002G255900	BB4-top	1	NAD(P)-binding Rossmann-fold superfamily protein
Potri.002G256001	BB4-top	1	
Potri.002G256100	BB4-top	1	
Potri.002G256200	BB4-top	1	Exostosin family protein
Potri.002G256300	BB4-top	1	Subtilase family protein
Potri.002G256400	BB4-top	1	photosystem II reaction center PSB28 protein
Potri.002G256500	BB4-top	1	Leucine-rich repeat transmembrane protein kinase family p
Potri.002G256600	BB4-top	1	indole-3-acetic acid inducible 11
Potri.002G256700	BB4-top	1	
Potri.002G258200	BB4-top	1	Leucine-rich repeat receptor-like protein kinase family pro
Potri.002G258300	BB4-top	1	

Potri.002G262100	BB4-top	1	
Potri.002G262200	BB4-top	1	
Potri.002G262300	BB4-top	1	GYF domain-containing protein
Potri.002G263450	BB4-top	1	
Potri.002G263500	BB4-top	1	
Potri.004G013100	BB4-top	1	Mitochondrial transcription termination factor family prote
Potri.004G013200	BB4-top	1	glyoxalase I homolog
Potri.004G013300	BB4-top	1	Pentatricopeptide repeat (PPR) superfamily protein
Potri.004G013400	BB4-top	1	arogenate dehydratase 1
Potri.004G162800	BB4-top	1	ATP binding microtubule motor family protein
Potri.004G162966	BB4-top	1	LYR family of Fe/S cluster biogenesis protein
Potri.004G163032	BB4-top	1	
Potri.004G163100	BB4-top	1	
Potri.004G163200	BB4-top	1	ubiquitin fusion degradation 1
Potri.010G035800	BB4-top	1	AAA-type ATPase family protein / ankyrin repeat family p
Potri.010G035900	BB4-top	1	peptidase M20/M25/M40 family protein
Potri.013G130400	BB4-top	1	Glycosyl hydrolase superfamily protein
Potri.013G130500	BB4-top	1	core-2/I-branching beta-1,6-N-acetylglucosaminyltransfera family protein
Potri.013G130600	BB4-top	1	Pentatricopeptide repeat (PPR) superfamily protein
Potri.013G130700	BB4-top	1	
Potri.017G006400	BB4-top	1	Pectin lyase-like superfamily protein
Potri.017G006500	BB4-top	1	Pectin lyase-like superfamily protein
Potri.018G041900	BB4-top	1	casein kinase I-like 3
Potri.018G041950	BB4-top	1	casein kinase I-like 3
Potri.018G042000	BB4-top	1	Plant protein of unknown function (DUF828) with plant pleckstrin homology-like region
Potri.018G042100	BB4-top	1	Glyoxalase/Bleomycin resistance protein/Dioxygenase superfamily protein
Potri.005G021400	BS7	1	eukaryotic translation initiation factor 3A
Potri.005G021500	BS7	1	
Potri.005G021601	BS7	1	PRP38 family
Potri.005G021700	BS7	1	TOPLESS-related 3
Potri.005G026400	BS7	1	RECQ helicase SIM
Potri.005G026450	BS7	1	
Potri.005G026500	BS7	1	histone
Potri.005G026600	BS7	1	Transducin/WD40 repeat-like superfamily protein
Potri.005G026700	BS7	1	soybean gene regulated by cold-2
Potri.013G000800	BS7	1	Major facilitator superfamily protein
Potri.013G000900	BS7	1	Pectinacetylesterase family protein
Potri.013G001000	BS7	1	myb domain protein 61
Potri.013G001150	BS7	1	neurofilament protein-related
Potri.013G001300	BS7	1	phytochrome interacting factor 3

Potri.013G003000	BS7	1	putative endonuclease or glycosyl hydrolase with C2H2-typ finger domain
Potri.013G003100	BS7	1	Putative endonuclease or glycosyl hydrolase
Potri.013G003200	BS7	1	Putative endonuclease or glycosyl hydrolase
Potri.016G006900	BS7	1	microtubule-associated protei
Potri.016G007000	BS7	1	
Potri.017G145901	BS7	1	Pectin lyase-like superfamily protein
Potri.017G146000	BS7	1	Protein kinase superfamily protein
Potri.017G146100	BS7	1	
Potri.017G146200	BS7	1	glutaminyl cyclase
Potri.017G146300	BS7	1	F-box/RNI-like superfamily
Potri.017G146400	BS7	1	F-box/RNI-like superfamily protein
Potri.010G043100	CO3-17	1	
Potri.010G043200	CO3-17	1	NSP-interacting kinase 3
Potri.010G043300	CO3-17	1	Plant protein of unknown function (DUF828)
Potri.010G043400	CO3-17	1	
Potri.010G162200	CO3-17	1	Glycosyltransferase family 61 protein
Potri.010G162300	CO3-17	1	chorismate mutase 3
Potri.010G162400	CO3-17	1	Protein of unknown function, DUF538
Potri.010G162500	CO3-17	1	Protein of unknown function (DUF155)
Potri.010G162600	CO3-17	1	homologue of bacterial
Potri.010G162700	CO3-17	1	Transducin/WD40 repeat-like superfamily
Potri.010G167100	CO3-17	1	RNA-binding KH domain-containing protein
Potri.010G167150	CO3-17	1	
Potri.010G167200	CO3-17	1	expansin A1
Potri.010G172900	CO3-17	1	Cation efflux family
Potri.010G173000	CO3-17	1	Uncharacterized conserved protein (DUF2358)
Potri.010G173100	CO3-17	1	Protein kinase superfamily protein
Potri.010G180000	CO3-17	1	HXXXD-type acyl-transferase family protein
Potri.010G180100	CO3-17	1	
Potri.010G180200	CO3-17	1	
Potri.010G180300	CO3-17	1	Cysteine proteinases superfamily protein
Potri.010G180400	CO3-17	1	2-phosphoglycolate phosphatase 1
Potri.010G180500	CO3-17	1	UDP-galactose transporter 6
Potri.010G184500	CO3-17	1	ferritin 2
Potri.010G184600	CO3-17	1	histidine acid phosphatase family protein
Potri.010G184700	CO3-17	1	PLAC8 family protein
Potri.010G184800	CO3-17	1	XH/XS domain-containing protein
Potri.010G184900	CO3-17	1	Eukaryotic translation initiation factor 2 subunit 1
Potri.010G185000	CO3-17	1	S-adenosyl-L-methionine-dependent methyltransferases superfamily protein
Potri.010G185100	CO3-17	1	Peptide-N4-(N-acetyl-beta-glucosaminyl)asparagine amida protein
Potri.010G185200	CO3-17	1	Ankyrin repeat family protein

Potri.010G205500	CO3-17	1	heat shock cognate prote
Potri.010G205600	CO3-17	1	Heat shock protein 70 (Hsp 70) family protein
Potri.010G205700	CO3-17	1	heat shock protein 70
Potri.012G070600	CO3-17	1	Protein of unknown function (DU
Potri.012G070700	CO3-17	1	nitrate transporter 1:2
Potri.012G070801	CO3-17	1	
Potri.013G118500	CO3-17	1	TRICHOME BIREFRINGENCE-LIKE 39
Potri.013G118600	CO3-17	1	late embryogenesis abundant domain-containing protei domain-containing
Potri.013G118700	CO3-17	1	UDP-glucosyl transferase 78D2
Potri.013G118800	CO3-17	1	
Potri.013G118900	CO3-17	1	global transcription factor g
Potri.013G147600	CO3-17	1	zinc ion binding
Potri.013G147700	CO3-17	1	Wound-responsive family protein
Potri.013G147750	CO3-17	1	
Potri.013G147800	CO3-17	1	PapD-like superfamily protein
Potri.013G147900	CO3-17	1	Wound-responsive family protein
Potri.013G148000	CO3-17	1	Wound-responsive family protein
Potri.004G228800	CO3-17, CO3-18	2	auxin response factor 1
Potri.004G228900	CO3-17, CO3-18	2	mitotic checkpoint family
Potri.002G013700	CO3-17, CO3-18, LS2-17, LS5-18	4	SKU5 similar 5
Potri.002G013800	CO3-17, CO3-18, LS2-17, LS5-18	4	enoyl-coA hydratase 2
Potri.002G013900	CO3-17, CO3-18, LS2-17, LS5-18	4	Prolyl oligopeptidase family protein
Potri.002G014000	CO3-17, CO3-18, LS2-17, LS5-18	4	Prolyl oligopeptidase family protein
Potri.002G014100	CO3-17, CO3-18, LS2-17, LS5-18	4	rho guanyl-nucleotide exchange factor 1
Potri.010G185800	CO3-17, CO8-17	2	UDP-glucose pyrophospho
Potri.010G185900	CO3-17, CO8-17	2	plant U
Potri.002G014200	CO3-17, LS5-18	2	
Potri.003G044800	CO3-18	1	
Potri.003G044900	CO3-18	1	AAA-type ATPase family protein
Potri.016G043500	CO3-18	1	INO80 ortholog
Potri.016G043600	CO3-18	1	Calcium-binding EF-hand family protein
Potri.016G043700	CO3-18	1	alpha carbonic anhydrase 4
Potri.016G043800	CO3-18	1	eukaryotic translation initiation factor 3E
Potri.002G037900	CO3-18, LS5-18	2	Peptidase S24/S26A/S26B/S26C family protein
Potri.002G038000	CO3-18, LS5-18	2	ENTH/VHS/GAT family protein
Potri.002G038100	CO3-18, LS5-18	2	RmlC-like cupins superfamily protein
Potri.002G038200	CO3-18, LS5-18	2	
Potri.002G038300	CO3-18, LS5-18	2	translocase inner membrane subunit 44-2
Potri.003G159500	CO3-18, LS5-18	2	Translation protein SH3-like family protein

Potri.003G159600	CO3-18, LS5-18	2	Protein phosphatase 2C family protein
Potri.003G159700	CO3-18, LS5-18	2	xyloglucan endotransglucosylase/hydrolase 5
Potri.003G159750	CO3-18, LS5-18	2	
Potri.007G077300	CO3-18, LS5-18	2	
Potri.007G077400	CO3-18, LS5-18	2	essential meiotic endonuclease 1B
Potri.007G077500	CO3-18, LS5-18	2	Protein of unknown function (DUF3511)
Potri.007G077600	CO3-18, LS5-18	2	Protein kinase superfamily protein
Potri.007G077700	CO3-18, LS5-18	2	
Potri.009G162601	CO8-17	1	nitrilase-like protein 1
Potri.009G162700	CO8-17	1	
Potri.009G162800	CO8-17	1	Protein of unknown function (DUF3049)
Potri.009G162900	CO8-17	1	Rho GTPase activating protein with PAK-box/P21-Rho-[illegible]
Potri.009G163001	CO8-17	1	
Potri.009G163100	CO8-17	1	copper ion binding
Potri.009G163200	CO8-17	1	copper/zinc superoxide dismutase 2
Potri.017G092100	CO8-17	1	TPX2 (targeting protein for Xklp2) protein family
Potri.017G092200	CO8-17	1	Ribosomal protein S19e family protein
Potri.017G093600	CO8-17	1	RS2-interacting KH protein
Potri.017G093650	CO8-17	1	exocyst subunit exo70 family protein E1
Potri.017G093700	CO8-17	1	exocyst subunit exo70 family protein E1
Potri.017G093800	CO8-17	1	exocyst subunit exo70 family protein E1
Potri.019G061800	CO8-17	1	serine/threonine protein kinase 2
Potri.019G061900	CO8-17	1	phosphate transporter 1;3
Potri.019G062000	CO8-17	1	thioredoxin H-type 9
Potri.019G062100	CO8-17	1	Leucine-rich repeat protein kinase family [illegible]
Potri.019G062200	CO8-17	1	
Potri.013G025900	CO8-17, LS5-17	2	basic helix-loop-helix (bHLH) DNA-binding superfamily [illegible]
Potri.013G026000	CO8-17, LS5-17	2	NAD(P)-binding Rossmann-fold superfamily protein
Potri.013G026100	CO8-17, LS5-17	2	NAD(P)-binding Rossmann-fold superfamily protein
Potri.001G137800	CO8-18	1	homeobox-7
Potri.002G203300	CO8-18	1	Protein of unknown function, DUF538
Potri.002G203400	CO8-18	1	Protein of unknown function, DUF538
Potri.002G230400	CO8-18	1	Late embryogenesis abundant (LEA) hydroxyproline-rich glycoprotein family
Potri.002G230500	CO8-18	1	DNA mismatch repair protein MutS, type 2
Potri.002G230600	CO8-18	1	Eukaryotic protein of unknown function (D[illegible]
Potri.019G129600	CO8-18	1	
Potri.019G129700	CO8-18	1	
Potri.019G129800	CO8-18	1	ChaC-like family protein
Potri.019G129900	CO8-18	1	chromatin remodeling 1
Potri.019G130000	CO8-18	1	Basic-leucine zipper (bZIP) transcription factor family [illegible]
Potri.009G106300	CO8-18, LS5-18	2	

Potri.009G106400	CO8-18, LS5-18	2	Peroxidase superfamily protein
Potri.009G106500	CO8-18, LS5-18	2	RNA-binding protein
Potri.001G392901	DBH17	1	UDP-Glycosyltransferase superfamily protein
Potri.001G393001	DBH17	1	
Potri.001G393101	DBH17	1	
Potri.006G031700	DBH17	1	disproportionating en
Potri.006G031800	DBH17	1	hydroxyproline-rich glycoprotein family protein
Potri.006G031900	DBH17	1	global transcription factor group B1
Potri.011G143700	DBH17	1	GTP-binding protein-
Potri.011G143800	DBH17	1	methyl esterase 14
Potri.011G143900	DBH17	1	
Potri.001G368300	LS2-17	1	concanavalin A-like lectin protein kinase family protein
Potri.001G368400	LS2-17	1	Polynucleotidyl transferase, ribonuclease H-like superfami protein
Potri.001G368500	LS2-17	1	NOL1/NOP2/sun family protein / antitermination NusB do containing protein
Potri.001G373200	LS2-17	1	GroES-like zinc-binding alcohol dehydrogenase family pro
Potri.001G373300	LS2-17	1	sequence-specific DNA binding transcription factors;seque specific DNA binding
Potri.001G373400	LS2-17	1	
Potri.001G374500	LS2-17	1	alpha/beta-Hydrolases superfamily protein
Potri.001G374600	LS2-17	1	glutamate receptor 2.7
Potri.001G375300	LS2-17	1	sodium/calcium exchanger family protein / calcium-bindin hand family protein
Potri.001G375400	LS2-17	1	sodium/calcium exchanger family protein / calcium-bindin hand family protein
Potri.001G375700	LS2-17	1	IQ-dor
Potri.001G375800	LS2-17	1	TCP family transcription factor 4
Potri.001G375900	LS2-17	1	P-loop containing nucleoside triphosphate hydrolases super protein
Potri.001G376900	LS2-17	1	
Potri.001G377000	LS2-17	1	Major facilitator superfamily protein
Potri.001G377100	LS2-17	1	
Potri.001G377200	LS2-17	1	DNA-directed RNA polymerase II
Potri.001G377300	LS2-17	1	
Potri.001G377400	LS2-17	1	RNA-binding (RRM/RBD/RNP motifs) family protein
Potri.001G378000	LS2-17	1	RING/U-box superfamily protein
Potri.003G103100	LS2-17	1	
Potri.003G103200	LS2-17	1	TRAF-like family
Potri.003G103300	LS2-17	1	CLP protease P4
Potri.003G103400	LS2-17	1	Ankyrin repeat family protein
Potri.003G103500	LS2-17	1	NAC-like, activated by AP3/PI
Potri.003G103600	LS2-17	1	Amino acid permease family
Potri.003G111700	LS2-17	1	Phosphatidic acid phosphatase (PAP2) family protein
Potri.006G214300	LS2-17	1	exocyst subunit exo70 family protein H4

Potri.006G214400	LS2-17	1	Pectin lyase-like superfamily protein
Potri.006G214500	LS2-17	1	Ankyrin repeat family
Potri.007G101100	LS2-17	1	ssDNA-binding transcriptional re
Potri.007G101201	LS2-17	1	wound-responsive family protein
Potri.007G101300	LS2-17	1	
Potri.009G021400	LS2-17	1	HSP20-like chaperones superfamily protein
Potri.009G021500	LS2-17	1	calmodulin 5
Potri.009G021600	LS2-17	1	5\'-AMP-activated protein kinase beta-2 subunit protein
Potri.013G100600	LS2-17	1	Nuclear transport factor 2 (NTF2) family
Potri.016G033300	LS2-17	1	Transcription elongation factor (TFIIS) family protein
Potri.016G033800	LS2-17	1	DNA/RNA helicase protein
Potri.016G033900	LS2-17	1	DNA/RNA helicase protein
Potri.016G034000	LS2-17	1	DNA/RNA helicase protein
Potri.016G034100	LS2-17	1	IQ calmodulin-binding motif family protein
Potri.016G034200	LS2-17	1	AtGCP3 interacting pr
Potri.016G034300	LS2-17	1	serine carboxypeptidase-like 45
Potri.016G033400	LS2-17, LS2-18	2	
Potri.016G033500	LS2-17, LS2-18	2	DNA/RNA helicase protein
Potri.016G033600	LS2-17, LS2-18	2	DNA/RNA helicase protein
Potri.016G033700	LS2-17, LS2-18	2	DNA/RNA helicase protein
Potri.003G213800	LS2-18	1	fatty acid desaturase 2
Potri.003G213900	LS2-18	1	
Potri.003G214000	LS2-18	1	C2H2 and C2HC zinc fingers superfamily
Potri.003G214100	LS2-18	1	Transducin/WD40 repeat-like superfamily protein
Potri.003G214200	LS2-18	1	glucan synthase-
Potri.006G232700	LS2-18	1	Protein phosphatase 2C family protein
Potri.006G232800	LS2-18	1	
Potri.006G232900	LS2-18	1	gamma vacuolar processing enzyme
Potri.006G233000	LS2-18	1	
Potri.010G111900	LS2-18	1	P-loop containing nucleoside triphosphate hydrolases supe
Potri.010G112000	LS2-18	1	nudix hydrolase homolog 25
Potri.010G112050	LS2-18	1	
Potri.001G034100	LS5-17	1	fucosyltransferase 1
Potri.001G034200	LS5-17	1	Ubiquitin-conjugating enzyme/RWD-like protein
Potri.001G034300	LS5-17	1	global transcription factor group B1
Potri.001G155700	LS5-17	1	Integrase-type DNA-binding superfamily protein
Potri.001G155750	LS5-17	1	
Potri.001G205700	LS5-17	1	CD2-binding protein-related
Potri.001G253000	LS5-17	1	SELT-like protein precursor
Potri.001G253100	LS5-17	1	Protein of unknown function (DUF803)
Potri.001G253200	LS5-17	1	3\'-5\'-exoribonuclease family protein

Potri.001G253700	LS5-17	1	phytochromobilin:ferredoxin oxidoreductase, chlor phytochromobilin synthase
Potri.001G253800	LS5-17	1	sugar transporter 14
Potri.001G253900	LS5-17	1	NAD(P)-binding Rossmann-fold superfamily protein
Potri.001G254001	LS5-17	1	indigoidine synthase A family protein
Potri.001G255200	LS5-17	1	
Potri.001G255300	LS5-17	1	
Potri.001G255400	LS5-17	1	RNA-binding (RRM/RBD/RNP motifs) family protein
Potri.001G255466	LS5-17	1	
Potri.001G255532	LS5-17	1	Zinc finger (CCCH-type) family protein / RNA recognitior (RRM)-containing protein
Potri.001G323500	LS5-17	1	B-box type zinc finger protein with CCT domain
Potri.001G323600	LS5-17	1	Endosomal targeting BRO1-like domain-containing protei
Potri.001G323700	LS5-17	1	Uncharacterised protein family (UPF0497)
Potri.001G323800	LS5-17	1	Esterase/lipase/thioesterase family
Potri.001G323900	LS5-17	1	
Potri.001G324000	LS5-17	1	Esterase/lipase/thioesterase family protein
Potri.001G324100	LS5-17	1	LRR and NB-ARC domains-containing disease resistance
Potri.001G324200	LS5-17	1	Esterase/lipase/thioesterase family protein
Potri.001G369100	LS5-17	1	cellulose synthase
Potri.001G369200	LS5-17	1	Plant protein of unknown function (DUF827)
Potri.001G387600	LS5-17	1	SecY protein transport family protein
Potri.002G263800	LS5-17	1	cytochrome P450, family 721, subfamily A, polypeptide 1
Potri.002G263900	LS5-17	1	Plant self-incompatibility protein S1 family
Potri.002G264000	LS5-17	1	
Potri.003G087600	LS5-17	1	S-adenosyl-L-methionine-dependent methyltransferases superfamily protein
Potri.005G085600	LS5-17	1	Domain of unknown function (DUF1726) ;Putative (D
Potri.005G085700	LS5-17	1	Tetratricopeptide repeat (TPR)-like superfamily protein
Potri.005G085800	LS5-17	1	violaxanthin de-epoxidase-related
Potri.005G085900	LS5-17	1	S-adenosylmethionine c
Potri.005G095900	LS5-17	1	potassium transporter 1
Potri.005G096000	LS5-17	1	
Potri.005G096100	LS5-17	1	
Potri.005G096500	LS5-17	1	
Potri.005G151000	LS5-17	1	
Potri.005G151100	LS5-17	1	Zinc knuckle (CCHC-type) family protein
Potri.006G200150	LS5-17	1	
Potri.006G200200	LS5-17	1	RING-H2 group F2A
Potri.007G018200	LS5-17	1	
Potri.007G018300	LS5-17	1	
Potri.007G018350	LS5-17	1	
Potri.007G018400	LS5-17	1	Cytochrome P450 superfamily protein

Potri.007G045100	LS5-17	1	Subtilisin-like serine endopeptidase family protein
Potri.010G180800	LS5-17	1	Calcineurin-like metallo-phosphoesterase superfamily
Potri.010G180900	LS5-17	1	GDSL-like Lipase/Acylhydrolase family
Potri.010G181000	LS5-17	1	Integrase-type DNA-binding superfamily protein
Potri.012G034300	LS5-17	1	little
Potri.012G034400	LS5-17	1	
Potri.012G034500	LS5-17	1	
Potri.012G034600	LS5-17	1	disease resistance family protein / LRR family protein
Potri.012G034700	LS5-17	1	FAD-binding Berberine family protein
Potri.012G034800	LS5-17	1	Nucleoporin interacting Component (Nup93/Nic96-like) fa protein
Potri.012G035200	LS5-17	1	
Potri.012G035300	LS5-17	1	SLAC1 homologue 3
Potri.012G077400	LS5-17	1	Xanthine/uracil permease family protein
Potri.012G105000	LS5-17	1	Polynucleotide adenylyltransferase family protein
Potri.012G105100	LS5-17	1	phosphate transporter 3;1
Potri.012G105200	LS5-17	1	AAA-type ATPase family protein
Potri.012G105300	LS5-17	1	acyl carrier protein 4
Potri.013G033000	LS5-17	1	Esterase/lipase/thioesterase family protein
Potri.013G033101	LS5-17	1	Esterase/lipase/thioesterase family protein
Potri.013G033200	LS5-17	1	Matrixin family
Potri.013G044100	LS5-17	1	Signal transduction histidine kinase, hybrid-type, ethylene
Potri.013G044200	LS5-17	1	
Potri.013G044300	LS5-17	1	Methyltransferase family protein
Potri.013G044400	LS5-17	1	
Potri.013G060300	LS5-17	1	calmodulin-binding protein
Potri.013G090400	LS5-17	1	WRKY DNA-binding protein 55
Potri.013G090500	LS5-17	1	Protein kinase family protein with ARM repeat
Potri.013G090800	LS5-17	1	SNF1 kinase hom
Potri.013G098700	LS5-17	1	Protein kinase family protein with ARM repeat
Potri.015G009566	LS5-17	1	ARABIDOPSIS TRITHORAX-RELATED PRO
Potri.015G009632	LS5-17	1	ARABIDOPSIS TRITHORAX-RELATED PROTEIN 6
Potri.015G009700	LS5-17	1	Transducin/WD40 repeat-like superfamily
Potri.015G048900	LS5-17	1	myosin heavy chain-related
Potri.015G049000	LS5-17	1	soluble N-ethylmaleimide-sensitive factor adaptor pro
Potri.015G049100	LS5-17	1	glucuron
Potri.015G050200	LS5-17	1	NAD(P)-binding Rossmann-fold superfamily
Potri.015G050300	LS5-17	1	Transmembrane Fragile-X-F-associated protein
Potri.015G053100	LS5-17	1	actin-related protein 4
Potri.015G053300	LS5-17	1	Eukaryotic aspartyl protease family protein
Potri.015G053400	LS5-17	1	aluminum-activated malate transporter 9
Potri.016G098100	LS5-17	1	C2H2-type zinc finger family protein

Potri.019G063400	LS5-17	1	FAD-binding Berberine family protein
Potri.019G087700	LS5-17	1	somatic embryogenesis receptor-like k
Potri.019G087800	LS5-17	1	
Potri.019G087900	LS5-17	1	SU(VAR)3-9 homolog 1
Potri.019G088000	LS5-17	1	Integrase-type DNA-binding superfamily protein
Potri.019G088100	LS5-17	1	SEC6
Potri.002G263600	LS5-17, BB4-top	2	indeterminate(ID)-do
Potri.002G263700	LS5-17, BB4-top	2	
Potri.003G123600	LS5-18	1	P-loop containing nucleoside triphosphate hydrolases super protein
Potri.003G123700	LS5-18	1	UDP-D-glucose/UDP-D-galactose 4-epimerase 1
Potri.003G123800	LS5-18	1	Homeodomain-like protein
Potri.003G123900	LS5-18	1	Transcription initiation factor TFIIE, beta subunit
Potri.003G131500	LS5-18	1	Nuclear transport factor 2 (NTF2) family protein
Potri.003G131550	LS5-18	1	
Potri.003G141200	LS5-18	1	Glycine-rich protein family
Potri.003G141300	LS5-18	1	SGNH hydrolase-type esterase superfamily protein
Potri.003G141400	LS5-18	1	Calcium-binding EF-hand family protein
Potri.003G141500	LS5-18	1	Galactose-binding
Potri.003G141600	LS5-18	1	Nuclear transport factor 2 (NTF2) family protein
Potri.003G141700	LS5-18	1	
Potri.007G076700	LS5-18	1	ribonucleotide reductase 1
Potri.007G076900	LS5-18	1	26S proteasome regulatory subunit, putative (RPN5)
Potri.009G049700	LS5-18	1	THUMP domain-containing protein
Potri.009G049800	LS5-18	1	HSP20-like chaperones superfamily protein
Potri.009G049900	LS5-18	1	HSP20-like chaperones superfamily protein
Potri.009G050000	LS5-18	1	hexokinase-like 1
Potri.009G092200	LS5-18	1	SWIB/MDM2 domain superfamily protein
Potri.009G092300	LS5-18	1	arabinogalactan protein 18
Potri.009G092400	LS5-18	1	Protein of unknown function (DUF620)
Potri.009G106200	LS5-18	1	peptidyl-prolyl cis-trans isomerase / cyclophilin-40 (CYP4 rotamase
Potri.009G049900	LS5-18	1	HSP20-like chaperones superfamily protein
Potri.009G050000	LS5-18	1	hexokinase-like 1
Potri.009G092200	LS5-18	1	SWIB/MDM2 domain superfamily protein
Potri.009G092300	LS5-18	1	arabinogalactan protein 18
Potri.009G092400	LS5-18	1	Protein of unknown function (DUF620)
Potri.009G106200	LS5-18	1	peptidyl-prolyl cis-trans isomerase / cyclophilin-40 (CYP4 rotamase

Table S11: Shared candidate genes between our results and Evans et al. (2014) and McKown et al. (2014).

Gene	Trait in our results	Source	Trait in source	Year	Description of function in Arabidopis
Potri.001G137800	CO8-18	Evans et al. (2014)	budset	NA	homeobox-7

Potri.001G155700	LS5-17	Evans et al. (2014)	bud burst	NA	Integrase-type DNA-binding superfamily protein
Potri.001G155700	LS5-17	Evans et al. (2014)	height	NA	Integrase-type DNA-binding superfamily protein
Potri.001G205200	BB4-stb	Evans et al. (2014)	budset	NA	Auxin efflux carrier family protein
Potri.001G253800	LS5-17	Evans et al. (2014)	height	NA	sugar transporter 14
Potri.001G255400	LS5-17	Evans et al. (2014)	budset	NA	RNA-binding (RRM/RBD/RNP motifs) family protein
Potri.001G275800	BB4-brn	Evans et al. (2014)	budset	NA	Calcium-dependent lipid-binding (CaLB domain) family protein
Potri.001G275900	BB4-brn	Evans et al. (2014)	budset	NA	RING/U-box superfamily protein
Potri.001G323800	LS5-17	Evans et al. (2014)	bud burst	NA	Esterase/lipase/thioesterase family protein
Potri.001G323800	LS5-17	Evans et al. (2014)	height	NA	Esterase/lipase/thioesterase family protein
Potri.001G324000	LS5-17	Evans et al. (2014)	bud burst	NA	Esterase/lipase/thioesterase family protein
Potri.001G369100	LS5-17	Evans et al. (2014)	bud burst	NA	cellulose synthase like E1
Potri.001G369100	LS5-17	Evans et al. (2014)	height	NA	cellulose synthase like E1
Potri.001G373300	LS2-17	Evans et al. (2014)	budset	NA	sequence-specific DNA binding transcription factors;sequence-specific DNA binding
Potri.001G373400	LS2-17	Evans et al. (2014)	budset	NA	
Potri.001G375400	LS2-17	Evans et al. (2014)	budset	NA	sodium/calcium exchanger family protein / calcium-binding EF hand family protein
Potri.001G375800	LS2-17	Evans et al. (2014)	bud burst	NA	TCP family transcription factor 4
Potri.001G375900	LS2-17	Evans et al. (2014)	bud burst	NA	P-loop containing nucleoside triphosphate hydrolases superfamily protein
Potri.001G377100	LS2-17	Evans et al. (2014)	bud burst	NA	
Potri.001G377200	LS2-17	Evans et al. (2014)	bud burst	NA	DNA-directed RNA polymerase II
Potri.001G387600	LS5-17	Evans et al. (2014)	budset	NA	SecY protein transport family protein
Potri.002G038100	CO3-18, LS5-18	Evans et al. (2014)	bud burst	NA	RmlC-like cupins superfamily protein
Potri.002G038100	CO3-18, LS5-18	Evans et al. (2014)	height	NA	RmlC-like cupins superfamily protein
Potri.002G112700	BB4-18	Evans et al. (2014)	height	NA	homolog of yeast ergosterol28
Potri.002G113000	BB4-18	Evans et al. (2014)	budset	NA	alpha/beta-Hydrolases superfamily protein
Potri.002G230600	CO8-18	Evans et al. (2014)	bud burst	NA	Eukaryotic protein of unknown function (DUF914)
Potri.002G230600	CO8-18	Evans et al. (2014)	budset	NA	Eukaryotic protein of unknown function (DUF914)

Potri.002G253200	BB4-top	Evans et al. (2014)	bud burst	NA	CLIP-associated protein
Potri.002G255300	BB4-top	Evans et al. (2014)	budset	NA	Tetratricopeptide repeat (TPR)-like superfamily protein
Potri.002G255400	BB4-top	Evans et al. (2014)	budset	NA	RPM1 interacting protein 13
Potri.002G255400	BB4-top	Evans et al. (2014)	height	NA	RPM1 interacting protein 13
Potri.002G256100	BB4-top	Evans et al. (2014)	height	NA	
Potri.002G256200	BB4-top	Evans et al. (2014)	budset	NA	Exostosin family protein
Potri.002G256600	BB4-top	Evans et al. (2014)	budset	NA	indole-3-acetic acid inducible 11
Potri.002G258300	BB4-top	Evans et al. (2014)	bud burst	NA	
Potri.002G262200	BB4-top	Evans et al. (2014)	bud burst	NA	
Potri.002G263500	BB4-top	Evans et al. (2014)	bud burst	NA	
Potri.002G263700	LS5-17, BB4-top	Evans et al. (2014)	budset	NA	
Potri.002G263800	LS5-17	Evans et al. (2014)	bud burst	NA	cytochrome P450, family 721, subfamily A, polypeptide 1
Potri.002G263900	LS5-17	Evans et al. (2014)	bud burst	NA	Plant self-incompatibility protein S1 family
Potri.002G264000	LS5-17	Evans et al. (2014)	bud burst	NA	
Potri.003G044800	CO3-18	Evans et al. (2014)	bud burst	NA	
Potri.003G044800	CO3-18	Evans et al. (2014)	height	NA	
Potri.003G111700	LS2-17	Evans et al. (2014)	bud burst	NA	Phosphatidic acid phosphatase (PAP2) family protein
Potri.003G111700	LS2-17	Evans et al. (2014)	height	NA	Phosphatidic acid phosphatase (PAP2) family protein
Potri.003G123700	LS5-18	Evans et al. (2014)	bud burst	NA	UDP-D-glucose/UDP-D-galactose 4-epimerase 1
Potri.003G131500	LS5-18	Evans et al. (2014)	budset	NA	Nuclear transport factor 2 (NTF2) family protein
Potri.003G141600	LS5-18	Evans et al. (2014)	budset	NA	Nuclear transport factor 2 (NTF2) family protein
Potri.003G141600	LS5-18	Evans et al. (2014)	height	NA	Nuclear transport factor 2 (NTF2) family protein
Potri.003G214200	LS2-18	Mckown et al. (2014)	100% Yellowing	2010	glucan synthase-like 12
Potri.003G214200	LS2-18	Mckown et al. (2014)	Branches	2009	glucan synthase-like 12
Potri.003G214200	LS2-18	Mckown et al. (2014)	Bud set	2009	glucan synthase-like 12
Potri.003G214200	LS2-18	Mckown et al. (2014)	Bud set	2010	glucan synthase-like 12
Potri.003G214200	LS2-18	Mckown et al. (2014)	Bud set	2008	glucan synthase-like 12
Potri.003G214200	LS2-18	Mckown et al. (2014)	Bud set*	2009	glucan synthase-like 12
Potri.003G214200	LS2-18	Mckown et al. (2014)	Growth period	2010	glucan synthase-like 12
Potri.003G214200	LS2-18	Mckown et al. (2014)	Height growth cessation	2009	glucan synthase-like 12

Potri.003G214200	LS2-18	Mckown et al. (2014)	Leaf drop	2008	glucan synthase-like 12
Potri.003G214200	LS2-18	Mckown et al. (2014)	Leaf drop	2009	glucan synthase-like 12
Potri.003G214200	LS2-18	Evans et al. (2014)	budset	NA	glucan synthase-like 12
Potri.004G013300	BB4-top	Evans et al. (2014)	height	NA	Pentatricopeptide repeat (PPR) superfamily protein
Potri.004G013400	BB4-top	Mckown et al. (2014)	Bud set	2010	arogenate dehydratase 1
Potri.004G013400	BB4-top	Mckown et al. (2014)	Bud set	2009	arogenate dehydratase 1
Potri.004G013400	BB4-top	Mckown et al. (2014)	Bud set*	2009	arogenate dehydratase 1
Potri.004G013400	BB4-top	Mckown et al. (2014)	Bud set*	2010	arogenate dehydratase 1
Potri.004G013400	BB4-top	Mckown et al. (2014)	Growth period	2010	arogenate dehydratase 1
Potri.004G013400	BB4-top	Mckown et al. (2014)	Height growth cessation	2009	arogenate dehydratase 1
Potri.004G013400	BB4-top	Mckown et al. (2014)	Leaf drop	2009	arogenate dehydratase 1
Potri.004G013400	BB4-top	Mckown et al. (2014)	Leaf drop	2008	arogenate dehydratase 1
Potri.004G013400	BB4-top	Mckown et al. (2014)	Post-bud set period	2010	arogenate dehydratase 1
Potri.004G013400	BB4-top	Evans et al. (2014)	budset	NA	arogenate dehydratase 1
Potri.004G163100	BB4-top	Evans et al. (2014)	budset	NA	
Potri.004G228900	CO3-17, CO3-18	Evans et al. (2014)	bud burst	NA	mitotic checkpoint family protein
Potri.005G095900	LS5-17	Mckown et al. (2014)	Bud set*	2009	potassium transporter 1
Potri.005G095900	LS5-17	Evans et al. (2014)	bud burst	NA	potassium transporter 1
Potri.005G096000	LS5-17	Evans et al. (2014)	bud burst	NA	
Potri.005G096100	LS5-17	Evans et al. (2014)	bud burst	NA	
Potri.005G143400	BB4-brn, BB4-stb	Evans et al. (2014)	budset	NA	Protein kinase superfamily protein
Potri.005G151100	LS5-17	Evans et al. (2014)	height	NA	Zinc knuckle (CCHC-type) family protein
Potri.005G151800	BB4-stb	Evans et al. (2014)	budset	NA	Di-glucose binding protein with Kinesin motor domain
Potri.006G060800	BB4-stb	Evans et al. (2014)	budset	NA	serine-rich protein-related
Potri.006G200200	LS5-17	Evans et al. (2014)	bud burst	NA	RING-H2 group F2A
Potri.006G200200	LS5-17	Evans et al. (2014)	budset	NA	RING-H2 group F2A
Potri.006G214400	LS2-17	Evans et al. (2014)	bud burst	NA	Pectin lyase-like superfamily protein
Potri.007G018300	LS5-17	Evans et al. (2014)	budset	NA	
Potri.007G018400	LS5-17	Evans et al. (2014)	height	NA	Cytochrome P450 superfamily protein
Potri.007G045100	LS5-17	Evans et al. (2014)	bud burst	NA	Subtilisin-like serine endopeptidase family protein
Potri.009G021500	LS2-17	Evans et al. (2014)	height	NA	calmodulin 5
Potri.009G050000	LS5-18	Evans et al. (2014)	budset	NA	hexokinase-like 1
Potri.009G092400	LS5-18	Evans et al. (2014)	budset	NA	Protein of unknown function (DUF620)

Potri.009G093500	BB2-stt	Evans et al. (2014)	budset	NA	1,2-alpha-L-fucosidases
Potri.009G095800	BB2-stt	Evans et al. (2014)	height	NA	GroES-like zinc-binding alcohol dehydrogenase family protein
Potri.009G162800	CO8-17	Evans et al. (2014)	bud burst	NA	Protein of unknown function (DUF3049)
Potri.010G016300	BB4-stb	Evans et al. (2014)	bud burst	NA	Raffinose synthase family protein
Potri.010G016400	BB4-stb	Evans et al. (2014)	height	NA	Raffinose synthase family protein
Potri.010G066900	BB4-stb	Evans et al. (2014)	bud burst	NA	proton gradient regulation 5
Potri.010G167200	CO3-17	Evans et al. (2014)	budset	NA	expansin A1
Potri.010G180000	CO3-17	Evans et al. (2014)	budset	NA	HXXXD-type acyl-transferase family protein
Potri.010G180100	CO3-17	Evans et al. (2014)	budset	NA	
Potri.010G180100	CO3-17	Evans et al. (2014)	height	NA	
Potri.010G180300	CO3-17	Evans et al. (2014)	budset	NA	Cysteine proteinases superfamily protein
Potri.010G180400	CO3-17	Evans et al. (2014)	budset	NA	2-phosphoglycolate phosphatase 1
Potri.010G180500	CO3-17	Evans et al. (2014)	budset	NA	UDP-galactose transporter 6
Potri.010G180800	LS5-17	Evans et al. (2014)	budset	NA	Calcineurin-like metallo-phosphoesterase superfamily protein
Potri.010G181000	LS5-17	Evans et al. (2014)	budset	NA	Integrase-type DNA-binding superfamily protein
Potri.010G181000	LS5-17	Evans et al. (2014)	height	NA	Integrase-type DNA-binding superfamily protein
Potri.012G070600	CO3-17	Evans et al. (2014)	bud burst	NA	Protein of unknown function (DUF1118)
Potri.012G070700	CO3-17	Evans et al. (2014)	bud burst	NA	nitrate transporter 1:2
Potri.012G077400	LS5-17	Evans et al. (2014)	budset	NA	Xanthine/uracil permease family protein
Potri.012G105000	LS5-17	Evans et al. (2014)	bud burst	NA	Polynucleotide adenylyltransferase family protein
Potri.012G105000	LS5-17	Evans et al. (2014)	budset	NA	Polynucleotide adenylyltransferase family protein
Potri.012G105200	LS5-17	Evans et al. (2014)	bud burst	NA	AAA-type ATPase family protein
Potri.013G001300	BS7	Evans et al. (2014)	budset	NA	phytochrome interacting factor 3
Potri.013G003100	BS7	Evans et al. (2014)	height	NA	Putative endonuclease or glycosyl hydrolase
Potri.013G003200	BS7	Evans et al. (2014)	budset	NA	Putative endonuclease or glycosyl hydrolase
Potri.013G026100	CO8-17, LS5-17	Evans et al. (2014)	bud burst	NA	NAD(P)-binding Rossmann-fold superfamily protein
Potri.013G026100	CO8-17, LS5-17	Evans et al. (2014)	budset	NA	NAD(P)-binding Rossmann-fold superfamily protein
Potri.013G026100	CO8-17, LS5-17	Evans et al. (2014)	height	NA	NAD(P)-binding Rossmann-fold superfamily protein

Potri.013G090400	LS5-17	Evans et al. (2014)	bud burst	NA	WRKY DNA-binding protein 55
Potri.013G090500	LS5-17	Evans et al. (2014)	bud burst	NA	Protein kinase family protein with ARM repeat domain
Potri.013G090800	LS5-17	Evans et al. (2014)	height	NA	SNF1 kinase homolog 10
Potri.013G094000	BB2-18	Evans et al. (2014)	budset	NA	S-locus lectin protein kinase family protein
Potri.013G094000	BB2-18	Evans et al. (2014)	height	NA	S-locus lectin protein kinase family protein
Potri.013G100600	LS2-17	Evans et al. (2014)	bud burst	NA	Nuclear transport factor 2 (NTF2) family protein
Potri.013G100600	LS2-17	Evans et al. (2014)	height	NA	Nuclear transport factor 2 (NTF2) family protein
Potri.013G118700	CO3-17	Evans et al. (2014)	bud burst	NA	UDP-glucosyl transferase 78D2
Potri.013G118800	CO3-17	Evans et al. (2014)	bud burst	NA	
Potri.013G118900	CO3-17	Evans et al. (2014)	bud burst	NA	global transcription factor group E4
Potri.013G130600	BB4-top	Evans et al. (2014)	bud burst	NA	Pentatricopeptide repeat (PPR) superfamily protein
Potri.013G130700	BB4-top	Evans et al. (2014)	bud burst	NA	
Potri.013G147800	CO3-17	Evans et al. (2014)	bud burst	NA	PapD-like superfamily protein
Potri.013G148000	CO3-17	Evans et al. (2014)	height	NA	Wound-responsive family protein
Potri.014G000100	BB4-stb	Evans et al. (2014)	height	NA	zinc ion binding;nucleic acid binding
Potri.015G049100	LS5-17	Mckown et al. (2014)	Leaf drop	2009	glucuronidase 2
Potri.015G049100	LS5-17	Evans et al. (2014)	budset	NA	glucuronidase 2
Potri.015G050300	LS5-17	Evans et al. (2014)	budset	NA	Transmembrane Fragile-X-F-associated protein
Potri.015G099200	BB4-18	Evans et al. (2014)	bud burst	NA	WRKY DNA-binding protein 75
Potri.015G099400	BB4-18	Evans et al. (2014)	bud burst	NA	
Potri.016G007000	BS7	Evans et al. (2014)	height	NA	
Potri.016G030000	BB4-18	Evans et al. (2014)	bud burst	NA	Leucine-rich repeat protein kinase family protein
Potri.016G030100	BB4-18	Evans et al. (2014)	bud burst	NA	syntaxin of plants 51
Potri.016G030200	BB4-18	Evans et al. (2014)	bud burst	NA	syntaxin of plants 52
Potri.016G030400	BB4-18	Evans et al. (2014)	bud burst	NA	Transducin/WD40 repeat-like superfamily protein
Potri.016G030500	BB4-17, BB4-18	Evans et al. (2014)	bud burst	NA	DNA/RNA helicase protein
Potri.016G030600	BB4-17, BB4-18	Evans et al. (2014)	bud burst	NA	DNA/RNA helicase protein
Potri.016G033300	LS2-17	Evans et al. (2014)	bud burst	NA	Transcription elongation factor (TFIIS) family protein
Potri.016G033300	LS2-17	Evans et al. (2014)	budset	NA	Transcription elongation factor (TFIIS) family protein
Potri.016G033400	LS2-17, LS2-18	Evans et al. (2014)	budset	NA	
Potri.016G033500	LS2-17, LS2-18	Evans et al. (2014)	budset	NA	DNA/RNA helicase protein

Potri.016G033600	LS2-17, LS2-18	Evans et al. (2014)	budset	NA	DNA/RNA helicase protein
Potri.016G043700	CO3-18	Evans et al. (2014)	bud burst	NA	alpha carbonic anhydrase 4
Potri.016G043800	CO3-18	Evans et al. (2014)	height	NA	eukaryotic translation initiation factor 3E
Potri.016G098100	LS5-17	Evans et al. (2014)	bud burst	NA	C2H2-type zinc finger family protein
Potri.016G098100	LS5-17	Evans et al. (2014)	height	NA	C2H2-type zinc finger family protein
Potri.016G137500	BB4-17	Evans et al. (2014)	bud burst	NA	VACUOLAR SORTING RECEPTOR 2
Potri.016G137500	BB4-17	Evans et al. (2014)	height	NA	VACUOLAR SORTING RECEPTOR 2
Potri.016G137700	BB4-17	Evans et al. (2014)	budset	NA	Lung seven transmembrane receptor family protein
Potri.016G137800	BB4-17	Evans et al. (2014)	budset	NA	Protein of unknown function, DUF599
Potri.017G092200	CO8-17	Evans et al. (2014)	bud burst	NA	Ribosomal protein S19e family protein
Potri.017G093600	CO8-17	Evans et al. (2014)	height	NA	RS2-interacting KH protein
Potri.017G148900	BB2-18	Evans et al. (2014)	budset	NA	Tic22-like family protein
Potri.018G008200	BB4-brn	Evans et al. (2014)	bud burst	NA	
Potri.018G082100	BB4-stt	Evans et al. (2014)	budset	NA	Protein of unknown function (DUF502)
Potri.018G082300	BB4-stt	Evans et al. (2014)	bud burst	NA	
Potri.018G082300	BB4-stt	Evans et al. (2014)	budset	NA	
Potri.019G063400	LS5-17	Evans et al. (2014)	bud burst	NA	FAD-binding Berberine family protein

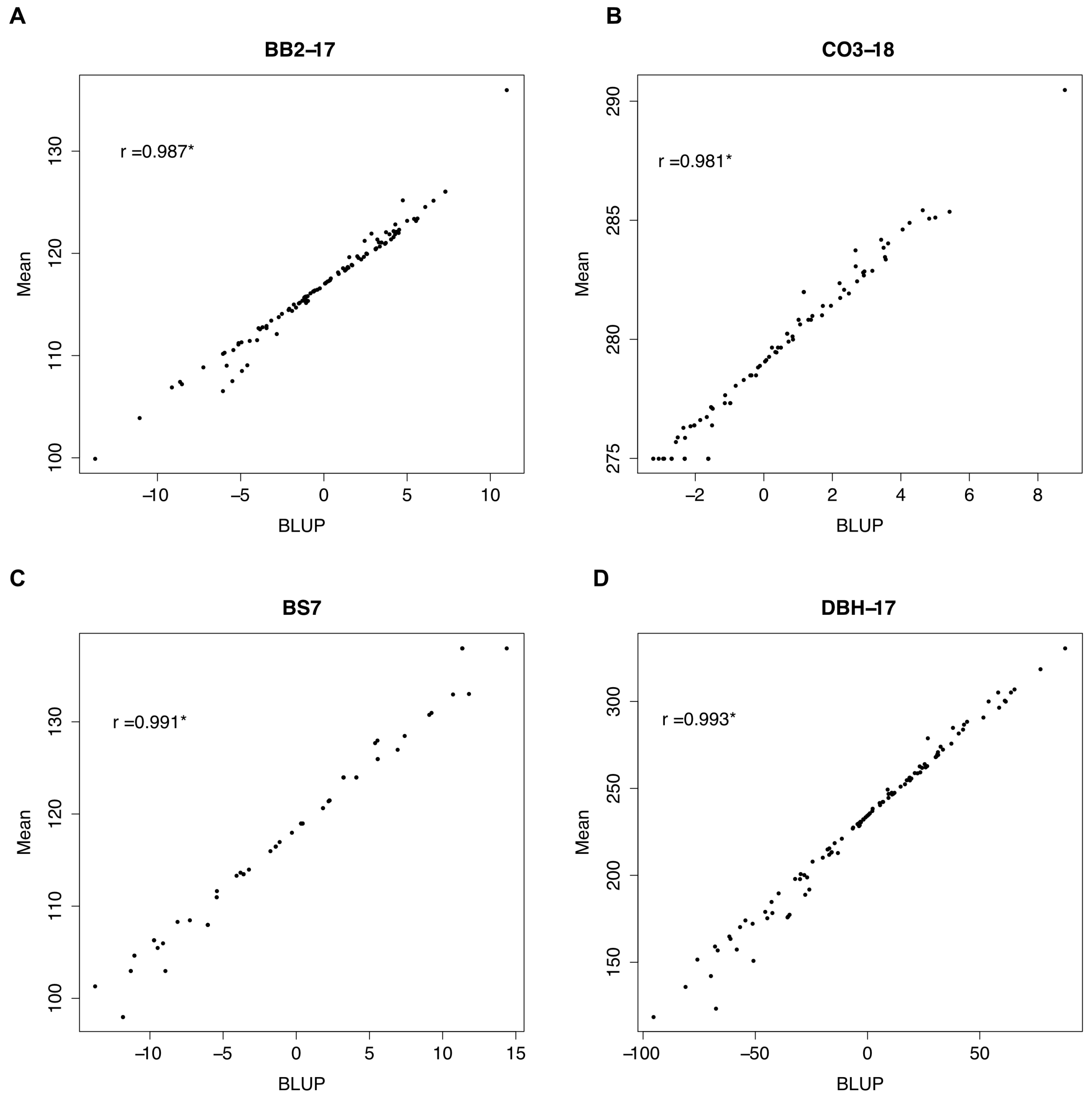

Figure S1: Pearson's correlation between BLUP and mean values of A) bud burst initation in year 2017 (BB2-17), B) autumn coloring initiation in year 2018 (CO3-18), C) bud set completion (BS7) and D) diameter at breast height (DBH-17). * $p < 2.2^{-16}$.

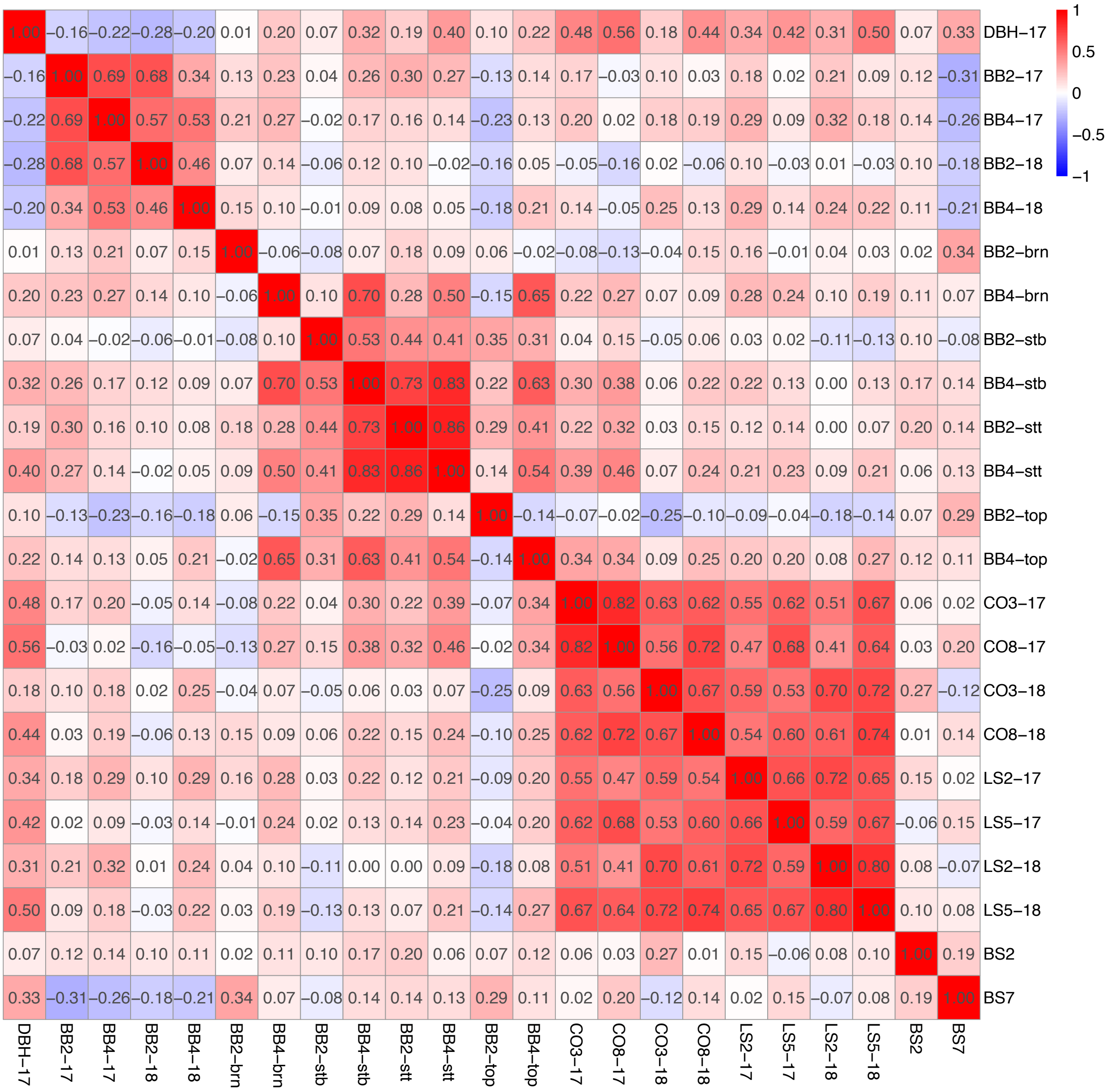

Figure S2: Spearman’s correlation matrix and heatmap of all our chosen 23 traits.

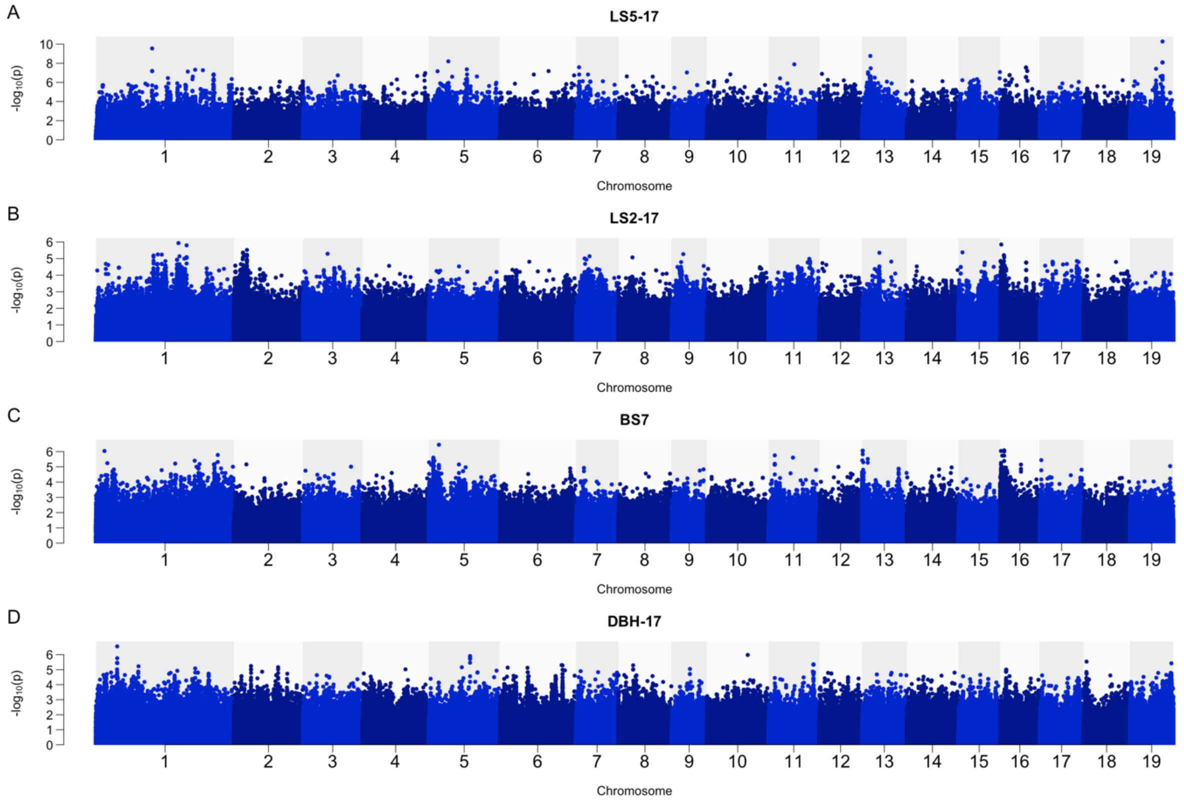

Figure S3: Logarithmic p-value Manhattan plot.

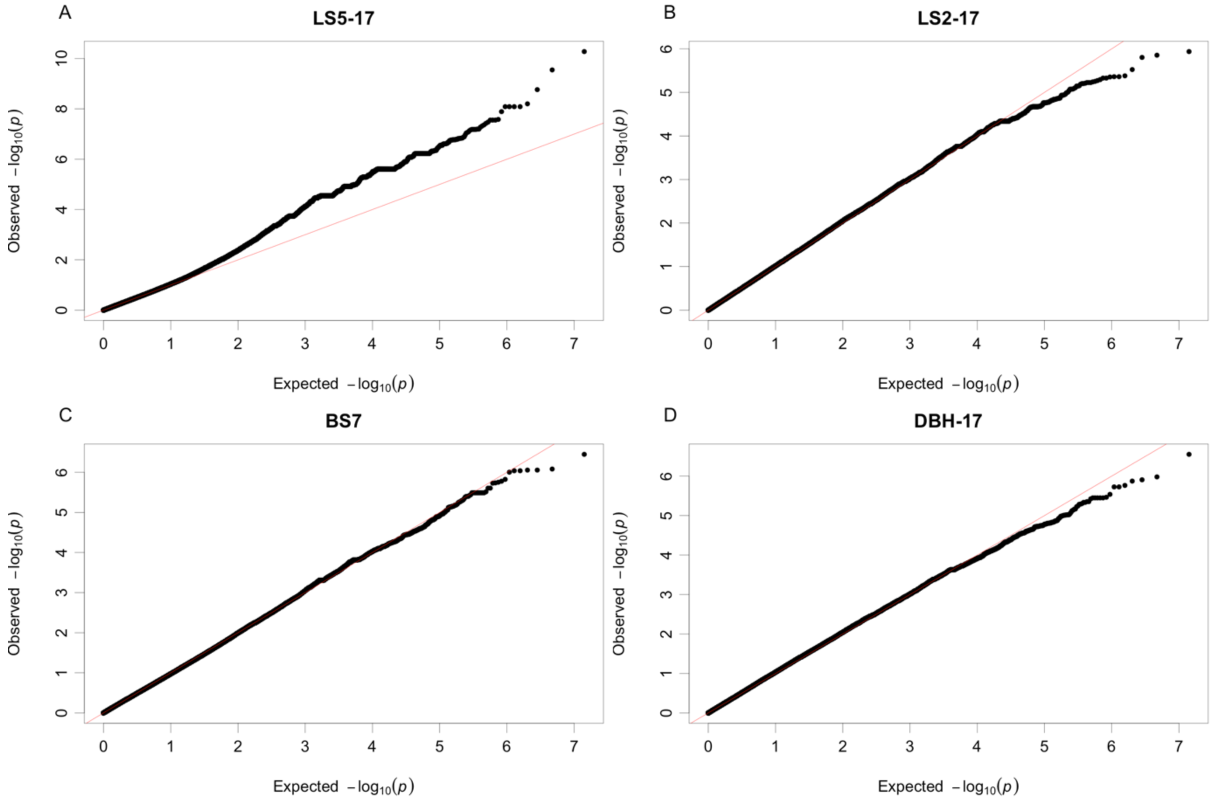

Figure S4: Logarithmic p-value QQ-plot.

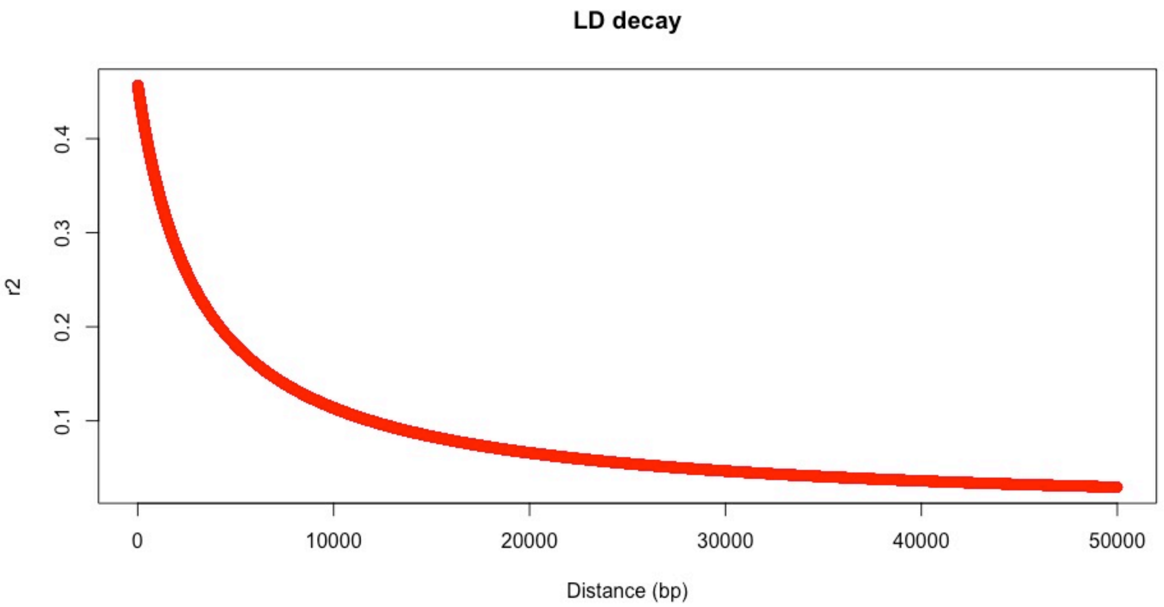

Figure S5: Level of LD as function of distance.

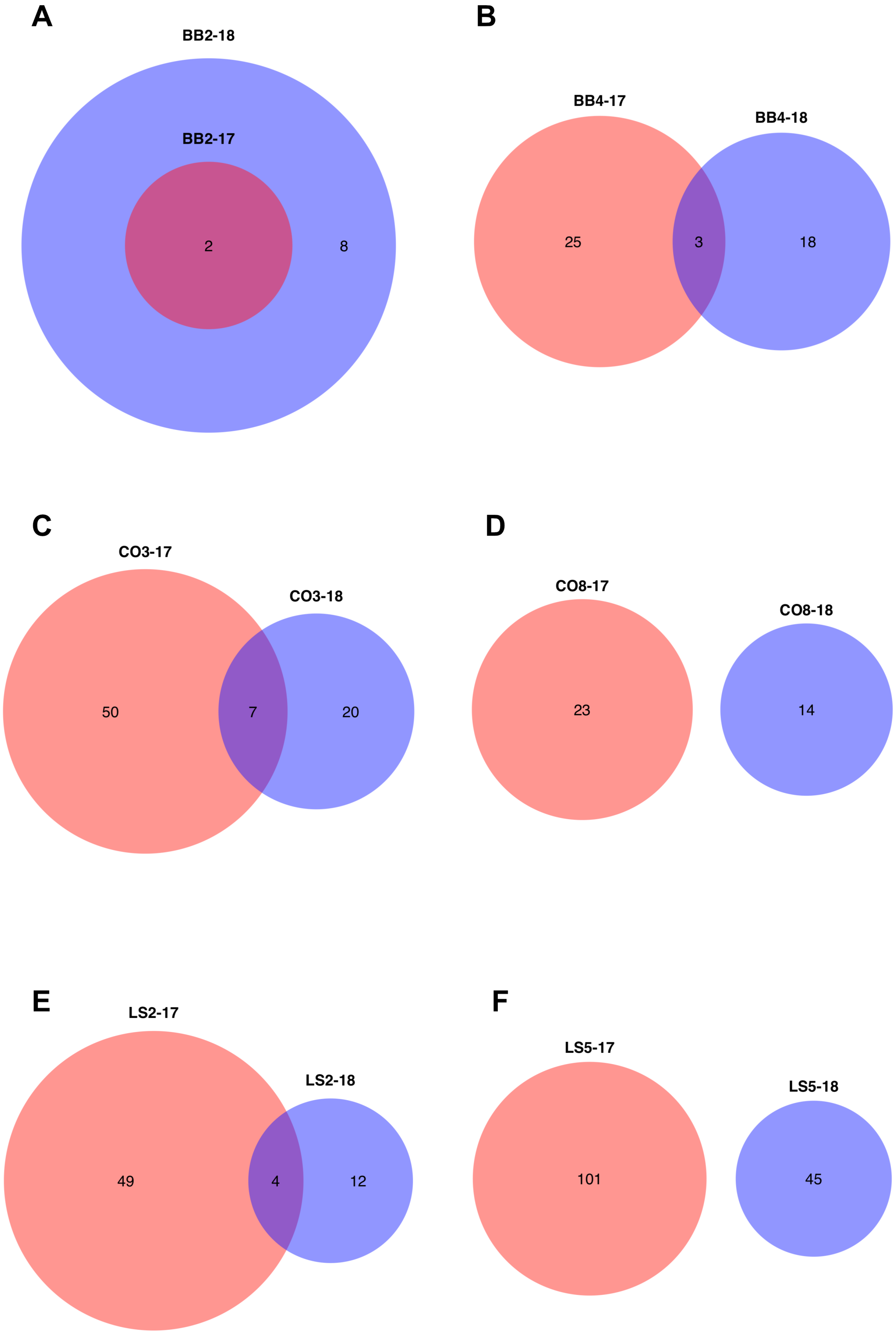
A
BB2-18
BB2-17
2
8
B
BB4-17
BB4-18
25
3
18
C
CO3-17
CO3-18
50
7
20
D
CO8-17
CO8-18
23
14
E
LS2-17
LS2-18
49
4
12
F
LS5-17
LS5-18
101
45

Figure S6: Venn diagram of candidate genes for both year for A) bud burst initiation (BB2), B) bud burs completion (BB4) C) autumn coloring initiation (CO3), D) autumn coloring completion (CO8) E) leaf shed initiation (LS2) and F) leaf shed completion (LS5).
